# Highly Stable and Reactive Platinum Single Atoms on Oxygen Plasma‐Functionalized CeO_2_ Surfaces: Nanostructuring and Peroxo Effects

**DOI:** 10.1002/anie.202112640

**Published:** 2022-03-16

**Authors:** Weiming Wan, Julian Geiger, Nikolay Berdunov, Mauricio Lopez Luna, See Wee Chee, Nathan Daelman, Núria López, Shamil Shaikhutdinov, Beatriz Roldan Cuenya

**Affiliations:** ^1^ Department of Interface Science Fritz Haber Institute Faradayweg 4–6 14195 Berlin Germany; ^2^ Institute of Chemical Research of Catalonia The Barcelona Institute of Science and Technology Institution 43007 Tarragona Spain

**Keywords:** CO Oxidation, Ceria, Plasma Functionalization, Single-Atom Catalysts, Surface Structures

## Abstract

Atomically dispersed precious metals on oxide supports have recently become increasingly interesting catalytic materials. Nonetheless, their non‐trivial preparation and limited thermal and environmental stability constitutes an issue for their potential applications. Here we demonstrate that an oxygen plasma pre‐treatment of the ceria (CeO_2_) surface serves to anchor Pt single atoms, making them active and resistant towards sintering in the CO oxidation reaction. Through a combination of experimental results obtained on well‐defined CeO_2_ films and theory, we show that the O_2_ plasma causes surface nanostructuring and the formation of surface peroxo (O_2_^2−^) species, favoring the uniform and dense distribution of isolated strongly bonded Pt^2+^ atoms. The promotional effect of the plasma treatment was further demonstrated on powder Pt/CeO_2_ catalysts. We believe that plasma functionalization can be applied to other metal/oxide systems to achieve tunable and stable catalysts with a high density of active sites.

## Graphical abstract

Pre‐treatment of the ceria (CeO_2_) surface with oxygen plasma assists the formation of highly dense and stable Pt single atoms resistant to sintering under CO oxidation conditions. The effect is assigned to plasma‐induced oxide nanostructuring and the formation of surface peroxo species which favor single‐atom metal adsorption. This approach can be utilized for the preparation of stable single‐atom catalysts with a high density of active sites.

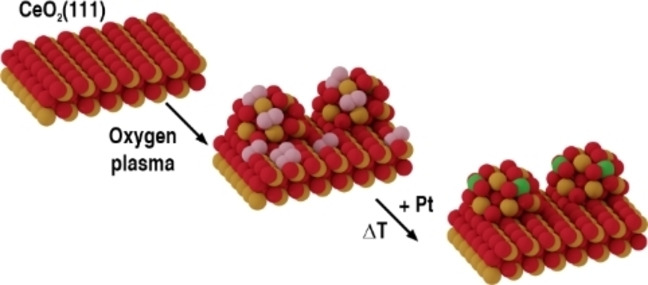

## Introduction

Single‐atom catalysts (SACs) of precious metals have recently received great attention in the catalysis community.^[1]^ In addition to the fact that SACs maximize the noble metal efficiency, such catalysts often show superior catalytic performance as compared to oxide‐supported metal nanoparticles (NPs). Several preparation techniques have been reported in the literature, such as co‐precipitation at very low metal loading,^[2]^ strong electrostatic adsorption of metal precursors on oxide nanocrystals,^[3]^ atom trapping,^[4]^ atomic layer deposition,^[5]^ magnetron co‐sputtering of a noble metal and an oxide,^[6]^ to name a few. To increase the commercial value of SACs, it is however necessary to fabricate them with relatively high metal loading. Moreover, single atoms must be resistant towards sintering under catalytically relevant conditions. To get more insight into these issues, fundamental studies employing model systems and surface sensitive techniques in combination with theoretical investigations are of high importance.

In this work, we addressed the question of how the surface structure (morphology and composition) of the oxide support affects the metal dispersion and the stability of single atoms at elevated temperatures and in a gas atmosphere. More specifically, we targeted ceria (CeO_2_)‐supported Pt SACs which are one of the most intensively studied systems, both experimentally and theoretically, in the CO oxidation reaction.^[7]^ We prepared Pt/CeO_2_ model catalysts by physical vapor deposition of Pt onto crystalline CeO_2_(111) films subjected to different pre‐treatments resulting in ceria reduction and/or surface roughening. Among the ceria supports studied, Pt atoms deposited onto CeO_2_ films pre‐treated with O_2_ plasma showed the best performance in terms of thermal stability and catalytic activity in the CO oxidation reaction. Corroborated by density functional theory (DFT), the effect is attributed to: i) a plasma‐induced surface restructuring resulting in ceria clusters at the oxide surface; and ii) the formation of a significant amount of surface peroxide (O_2_^2−^) species, all favoring a dense and uniform distribution of isolated and highly stable Pt single atoms. The promotional effect of the plasma treatement was also demonstrated on the powder catalysts. These findings open a new playground for using the plasma functionalization in heterogeneous catalysis to achieve a high density of stable active sites.

## Results and Discussion

Well‐ordered, stoichiometric CeO_2_(111) films were grown on a Ru(0001) substrate as described elsewhere^[8]^ (see the Experimental Section in the Supporting Information). To examine the possible role of oxygen vacancies on the Pt adsorption, the films were annealed in ultrahigh vacuum (UHV) at high temperature resulting in ceria partial reduction (henceforth referred to as “CeO_2_‐red” films). In addition, we prepared CeO_2_(111) films exposing a high density of monoatomic steps (“CeO_2_‐step”) as described in ref. [9] in order to increase the number of low‐coordinated sites at the surface. Finally, the well‐ordered films were treated with a low‐pressure oxygen plasma (denoted as “CeO_2_‐plasma”). Pt was deposited onto these films at low metal flux in order to minimize metal aggregation upon deposition at room temperature. The electronic structure of Pt was monitored by X‐ray Photoelectron Spectroscopy (XPS) via the Pt 4f level, and the binding energy (BE) of the 4f_7/2_ state will only be used in the discussion.

**Figure 1:**
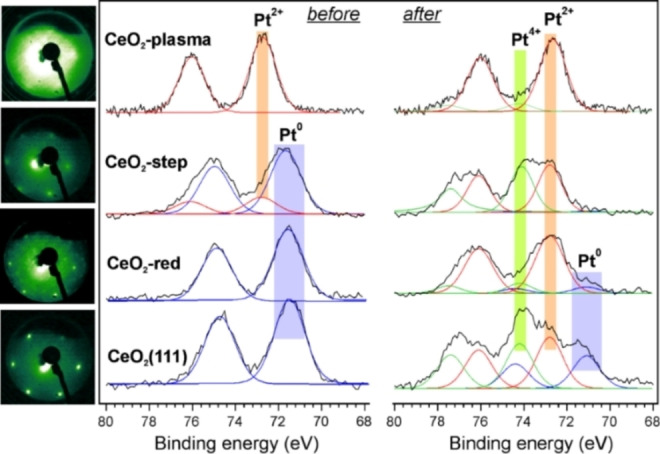
Comparison of Pt 4f spectra obtained on four different 0.2 ML Pt/CeO_2_ surfaces before and after CO oxidation reaction at 523 K (in a mixture of 10 mbar CO and 50 mbar O_2_ balanced by He to 1 bar). Before the reaction, the samples were annealed at 523 K in UHV. LEED patterns (at 81 eV) of the ceria films prior to the Pt deposition are shown in the left panel adjacent to the spectra.

Figure 1 compares Pt 4f spectra obtained for 0.2 ML Pt deposited on differently prepared CeO_2_ films (one monolayer (ML) corresponds to one Pt atom per CeO_2_(111) surface unit cell, i.e., 8×10^14^ cm^−2^) and measured before and after the CO oxidation reaction at 523 K at near ambient pressure. Note that all samples before the reaction were annealed in UHV at 523 K in order to discriminate solely thermal from reaction‐induced effects. The full XPS data set for the Pt 4f, Ce 3d and O 1s levels are shown in Figures S1–S4. The Low Energy Electron Diffraction (LEED) patterns of the films prior to the Pt deposition are shown in Figure 1 adjacent to the spectra.

First, we discuss the results for Pt deposited onto a well‐ordered stoichiometric CeO_2_(111) film. “As deposited” Pt species are characterized by a BE of 71.8 eV (Figure S1) which is usually observed on metallic Pt clusters.^[10]^ The peak shifts to lower BE (71.4 eV) upon UHV annealing, thus indicating particles sintering.^[11]^ The spectrum obtained after the reaction consists of several states. Obviously, the 71.1 eV state is associated with even larger metallic NPs, suggesting further Pt sintering under reaction conditions. Two other signals centered at 74.2 and 72.7 eV are assigned to Pt in the 4+ and 2+ oxidation states, respectively.

Analysis of the Ce 3d and O 1s spectra (Figure S1) revealed no changes after Pt deposition. The amount of Ce^3+^ slightly increases after vacuum annealing at 523 K, indicating a Pt‐induced partial ceria reduction. The Ce 3d spectrum fully recovers after CO oxidation in an O_2_‐rich atmosphere. The O 1s signal at 529.3 eV showed no additional states associated with adsorbates (typically, hydroxyls and carbonates) before and after Pt deposition, since the stoichiometric CeO_2_(111) surface is essentially inert to residual gases in the UHV background at 300 K.^[12]^ However, a considerable signal at 531.8 eV appears after the reaction, which can be attributed to adventitious CO_2_ (and probably water) adsorption on the bare ceria support at near ambient pressures.^[8b]^

The reduced ceria surface shows a complex ($\sqrt{7}$ ×$\sqrt{7}$ )‐R19.1° LEED pattern, which is assigned to long‐range ordering of the O vacancies at the surface.^[13]^ In contrast to stoichiometric CeO_2_(111), this surface readily reacts with traces of water in the UHV background causing an additional weak O 1s signal at 532.0 eV from surface hydroxyls (Figure S2).^[12b]^ Beyond the Pt metal atoms constituting small clusters (BE at 71.6 eV), some Pt atoms on the “CeO_2_‐red” surface are in the 2+ oxidation state (at 72.8 eV). However, the Pt^2+^ signal disappears upon vacuum annealing, whereas the Pt^0^ signal gains in intensity, so that the spectrum becomes virtually identical to the one observed on the stoichiometric CeO_2_ surface (Figure 1). After CO oxidation, Pt is found almost exclusively in the 2+ state (72.8 eV), with some minor contribution of Pt^4+^ (74.2 eV) and the Pt^0^ states (71.1 eV). Again, the results can be explained by the formation of relatively large Pt NPs which underwent oxidation. Concomitantly, the ceria surface became fully oxidized (no Ce^3+^ detected) in the O_2_‐rich mixture used (Figure S2).

When Pt is deposited onto CeO_2_(111) films with an artificially increased step density (“CeO_2_‐step”), the Pt atoms are partially in the 2+ and metallic states (BEs at 72.8 and 71.9 eV, respectively, Figure S3). In contrast to the previous cases, Pt^2+^ species are thermally stable and remain at the surface in addition to small metallic clusters, which sinter upon annealing (the corresponding BE shifts from 71.9 to 71.7 eV). After the CO oxidation reaction, no metallic Pt atoms are found on the surface, which now contains only Pt^2+^ and Pt^4+^ species.

Therefore, in the above‐presented systems, Pt deposits always form small metal particles upon UHV annealing. A certain amount of thermally stable Pt^2+^ species found on the “CeO_2_‐step” surface can be explained by the strong adsorption of Pt single atoms at step edges.^[9]^ When exposed to the CO oxidation atmosphere, metal clusters become oxidized, thus giving rise to the 4+ and 2+ states, albeit their ratio depends on the initial particle size. The formation of Pt^4+^ and Pt^2+^ species on the Pt NPs in oxidizing ambient is well‐documented in the literature,^[14]^ and is commonly attributed to PtO_2_/PtO clusters or a thin PtO_*x*_ oxide film formed on the large Pt NPs.

Finally, Figure 1 shows the results for the CeO_2_ film exposed to oxygen plasma prior to the Pt deposition. This system substantially differs from the previous ones. Starting from the “as deposited” sample (Figure S4), Pt remains exclusively in the 2+ state even in the CO oxidation reaction at near atmospheric pressure.

**Figure 2:**
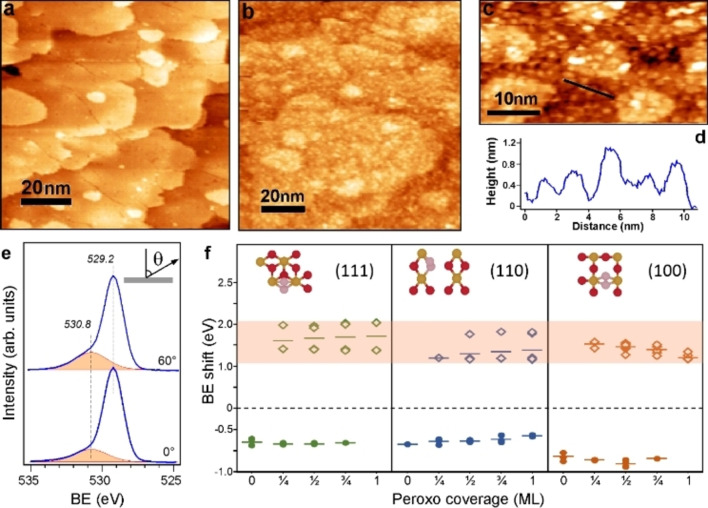
STM images of a) the well‐ordered CeO_2_(111) and b, c) CeO_2_(111)‐plasma films taken in UHV at ∼500 K. d) Topography profile along the line marked in image (c). e) O 1s XPS spectra of the CeO_2_(111)‐plasma film measured at normal and grazing emissions. f) Calculated BE shifts for the peroxo‐O atoms (open symbols) and surface O atoms (filled symbols) referenced to the lattice O atoms in the bulk (set to zero). The latter are in abundance and dominate the experimental spectra. The shaded area highlights experimentally measured BE shifts. Insets show the top views of the topmost layer in DFT‐optimized structures at 1/4 ML peroxo coverage on (111), (110), and (100) surfaces. Color code: Ce: gold; surface O: red; peroxo O_2_^2−^: pink.

To shed light on the origin of the exceptional stability of Pt on the CeO_2_‐plasma films, we studied the ceria surface with STM. STM images (Figure 2a–c) revealed the formation of ceria clusters about 1 nm in size, which are randomly distributed on the surface. These STM results also explain the lack of long‐range order in LEED patterns (see Figure 1). In addition, the O 1s XPS spectra (Figure 2e) revealed a prominent shoulder at 530.8 eV, which cannot be assigned to adventitious hydroxyls and/or carbonates, showing a considerably higher BE, i.e., 531.8 eV (Figure S1–S3). The signal is shifted by 1.6 eV with respect to the main peak at 529.2 eV and grows at increasing plasma exposure time. The signal gains in intensity when measured at more surface sensitive, grazing emission (Figure 2e). All these findings point to the formation of new oxygen species which are absent on other ceria surfaces studied above. Based on DFT (PBE + U) calculations of atomic O adsorption on the stoichiometric CeO_2_(111) surface and corresponding BE shifts of the O 1s level (Figure 2f, see details below), we assigned the 530.8 eV signal to surface peroxides, O_2_^2−^ (see also Figure S6, S7 and Table S1).

On such a disordered and very rough surface, direct visualization of metal single atoms with STM is very difficult if not impossible. In an attempt to identify Pt species formed on the CeO_2_‐plasma films, we employed Infrared Reflection‐Absorption Spectroscopy (IRAS) using CO as a probe molecule and compared the results on the Pt/CeO_2_‐plasma and pristine Pt/CeO_2_(111) surfaces. The bare ceria surfaces did not adsorb CO under the exposure conditions studied.

**Figure 3:**
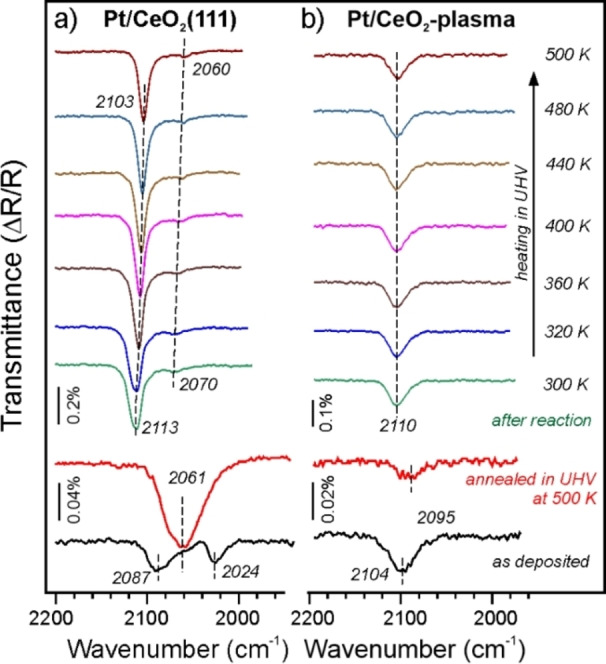
IRA‐spectra measured in UHV on 0.2 ML Pt deposited on CeO_2_(111) a) and CeO_2_‐plasma b) surfaces. CO was dosed at 200 K onto “as deposited” samples and after UHV annealing at 500 K for 5 min. The samples tested in the CO oxidation reaction were measured without additional CO dosage. The spectra were consecutively recorded while heating the sample in UHV to the temperatures indicated.

Adsorption of CO (at 10^−6^ mbar) on Pt deposited onto a well‐ordered CeO_2_(111) surface at 200 K (Figure 3a) resulted in IRAS bands centered at 2087 and 2024 cm^−1^ and a weak band as a shoulder at ∼2060 cm^−1^. The former two signals disappeared on the sample heated to 500 K and exposed to CO again, while the band at 2060 cm^−1^ gained in intensity. This latter band can be assigned to the stretching vibrations of CO adsorbed at the low‐coordinated sites^[15]^ on Pt NPs located primarily at the step edges, as shown by STM.^[11]^ Accordingly, the 2087 and 2024 cm^−1^ bands observed immediately after Pt deposition belong to CO adsorbed onto Pt clusters and aggregates with ill‐defined structures,^[16]^ since their formation is affected by the limited diffusivity of surface ad‐atoms at these relatively low temperatures. In contrast, Pt species formed upon deposition onto the CeO_2_‐plasma surface showed a single band at 2104 cm^−1^. The band is considerably reduced in intensity and red‐shifted (to 2095 cm^−1^) on the sample that was annealed in UHV at 500 K prior to the CO exposure (Figure 3b). The latter spectrum is fully reproducible after several thermal flashes to 500 K, indicating a good thermal stability of the Pt species, in full agreement with the XPS results. In principle, both the 2104 and 2095 cm^−1^ band falls in the range of those reported in the literature for powder Pt SACs proven by high resolution electron microscopy.[^1f^, ^4^, ^5^, ^7b^] It is interesting that no CO IRAS bands were observed on the samples pre‐heated to 700 K, indicating that either the Pt atoms migrate into the sub‐surface region or they adsorb CO weakly. It should be noted, however, that IRAS spectra on metal‐supported oxide films obey the selection rule such that only vibrations associated with dipole changes normal to the metal surface can be detected.^[17]^ Therefore, CO molecules oriented parallel to the Ru(0001) substrate will be invisible in IRAS spectra.

We also recorded the IRAS spectra on these two systems after the CO oxidation reaction in the mixture consisting of 1 % CO and 5 % O_2_ (balanced by Ar to 1 bar) in a “high‐pressure” reaction cell. After 5 min of reaction at 500 K and sample cooling to room temperature, the cell was pumped out and the IRAS spectra were recorded in UHV at 300 K without additional exposure to CO. Interestingly, the spectra revealed similar bands at around 2110 cm^−1^ on both systems, but of different intensities. (A weak and broad band at 2070 cm^−1^ on Pt/CeO_2_(111) can be assigned to traces of CO residing at the edges of metallic Pt NPs, see above). However, the spectra showed different behavior upon slow heating the sample to 500 K. On Pt/CeO_2_(111) (Figure 3a), the band at 2113 cm^−1^ gradually shifts to a lower wavenumber (by 10 cm^−1^), and its integral intensity slightly decreases, indicating a partial desorption of CO. These spectral changes and peak positions are characteristic for CO adsorbed on O‐precovered Pt surfaces,^[18]^ and the shift can be explained in terms of the dipole‐dipole interaction between neighboring CO molecules adsorbed on the partially oxidized Pt NPs, as observed by XPS (Figure 1). The formation of relatively large Pt NPs on CeO_2_(111) prior to the reaction can also explain the relatively high intensity of the IRAS bands due to the image charge effects. In contrast, a much weaker band at 2010 cm^−1^ observed on the Pt/CeO_2_‐plasma surface stays constant during sample heating to 500 K (Figure 3b). Although this band fully disappears upon heating to 700 K and cannot be observed upon CO exposure at room temperature, it is fully reproduced after a second CO oxidation reaction run. Based on the comparative XPS and IRAS results, we can conclude that Pt deposited onto the plasma‐treated CeO_2_ surface forms single atoms which remain stable in a reaction atmosphere, albeit its coordination to the ceria surface may be affected by the CO oxidation reaction.

Based on structural characterization of the CeO_2_‐plasma surface by LEED and STM, one may suggest that the enhanced stability of Pt originates from surface roughening that suppresses the diffusivity of the Pt ad‐atoms and hence their aggregation. To examine this scenario, we prepared a rough surface by bombarding a well‐ordered CeO_2_(111) film with 1 keV Ar^+^ ions at 300 K for 5 min, resulting in the strong attenuation of the diffraction spots in LEED and a high density of monatomic steps as previously shown by STM.^[19]^ However, the surface becomes considerably reduced due to the preferential sputtering of lighter O atoms. To re‐oxidize the ceria surface, the film was exposed to 10^−6^ mbar O_2_ at 500 K. Subsequent deposition of 0.2 ML Pt onto this “CeO_2_‐sputter” support resulted in Pt species primarily in the 2+ state (Figure S5a), but a considerable amount of Pt was also found in the metallic state (at 71.5 eV), most likely as Pt NPs. For comparison, the latter only appeared on Pt/CeO_2_‐plasma samples at a Pt coverage about two times higher, i.e., 0.4 ML. Apparently, the plasma creates more “O‐rich” sites to stabilize Pt single atoms. Indeed, a substantially higher intensity is observed for the peroxo‐related 530.8 eV signal in the O 1s spectra as compared to the CeO_2_‐sputter sample (Figure S5b).

Previous studies on Pt/CeO_2_ systems prepared by magnetron sputtering of Pt and CeO_2_ ^[6]^ and by co‐deposition of Ce and Pt in an oxygen atmosphere^[20]^ also reported exceptional stability of Pt^2+^ species, although the presence of Pt *at the surface* was not proven. DFT simulations on ceria clusters suggested a specific structural element named “nanopocket” that binds Pt^2+^ so strongly that it can withstand sintering and bulk diffusion.[^16^, ^20^] In these sites, Pt is coordinated to four oxygen atoms forming a planar Pt‐4O moiety. Such a coordination has also been identified on the (100)‐oriented surface and monoatomic step edges on the (111) surface.[^9^, ^20^, ^21^]

To get more insight into the interaction of the Pt atoms with the plasma‐treated CeO_2_ films, we investigated the systems at hand with DFT. First, we addressed the nature of surface oxygen species resulting from adsorption of oxygen radicals in the plasma^[22]^ on the low Miller‐index CeO_2_ surfaces. The O ad‐atoms readily react with lattice oxygen to form peroxo (O_2_^2−^) groups (see insets in Figure 2f, Figures S6, S7 and Tables S2–S6), in agreement with previous DFT studies of molecular O_2_ adsorption on partially reduced ceria surfaces.^[23]^ The computed BE shifts for the O_2_^2−^ groups depend on the coverage, and are 1.65, 1.30, and 1.35 eV, on average, for the (111), (110) and (100) surface, respectively (Figure 2f, Table S1). These values match well the BE shifts observed experimentally (Figure 2e), which can therefore be assigned to surface peroxo species in the plasma‐treated films. The above‐mentioned difference and similarity of the CeO_2_‐sputter and CeO_2_‐plasma surfaces may be linked to the mechanism by which peroxides are formed on the two surfaces. For the CeO_2_‐sputter samples, the oxygen vacancies, initially formed during Ar^+^ bombardment, react with molecular O_2_ in the re‐oxidation step forming the surface peroxide groups. However, oxygen vacancies on CeO_2_ may also migrate into subsurface positions^[23a]^ and hence become inaccessible to the reaction with O_2_. During the plasma treatment, however, molecular oxygen that is also present in the ambient immediately quenches the O vacancies (if formed) and ultimately results in surface peroxides in addition to those formed by direct reaction with atomic oxygen species in the plasma. As such, the higher ability of the oxygen‐plasma treated surface to stabilize Pt atoms could have its origin in the higher surface peroxide coverage, assuming that a similar nanostructuring is achieved with both treatments.

To understand the rationale behind the formation of ceria NPs observed by STM (Figure 2a–c) we considered a thermodynamic model^[24]^ under the assumption that the material forming the ceria NPs stems from the top CeO_2_(111) layers of the sample (Figure S10, Note S3). Consequently, the ceria film becomes thinner, while the total number of ceria formula units remains constant during the restructuring (Ce atoms are not sputtered by the relatively light O atoms in the plasma). We approximated a ceria NP as a hemisphere that grows in registry with the underlying film and employed surface energies computed for different peroxo‐covered extended surfaces (Tables S2–S6), as well as step and corner energies, using calculations of high Miller‐index surfaces (Tables S9, S10). Depending on the input parameters, the computed ceria NPs were between 3 and 11 nm in diameter (Figure S11), thus larger than experimentally observed. Nonetheless, these thermodynamic considerations demonstrate that the oxygen plasma definitely promotes surface restructuring and the formation of ceria NPs on the initially flat film, in addition to the possible bombardment effects of the high‐energy O atoms in the plasma. The increase in surface area leads to a larger number of local surface structures that can stabilize oxygen radicals via the peroxo groups, while the exposure of facets other than (111) on ceria NPs simultaneously entails a more exothermic reaction energy for the formation of peroxides.

Not surprisingly, peroxo species affect the Pt adsorption on the CeO_2_ surfaces (Table 1 and Figure S8). Compared to pristine CeO_2_(111), a Pt atom on the peroxo‐covered (111) surface is bound by 1–2 eV more strongly, albeit depending on the peroxide coverage. The effect is smaller for the (110) surface (the energy gain is about 0.6 eV, on average), while on the (100) surface, peroxo species may even weaken the Pt bonding. Among the surfaces studied, the strongest Pt adsorption is observed for (100)−4O coordination environment, in agreement with previous results.[^9^, ^20^] Interestingly, the presence of peroxo groups showed no beneficial effect in terms of Pt bonding for this site. We also examined Pt adsorption on stepped surfaces (Figure S9). Even though square‐planar 4O‐coordination environments, particularly on the (210) and (310) surfaces, provide sites for strongly exothermic Pt adsorption (Table S7), the stepped surfaces exhibit considerably higher surface energies and are therefore thermodynamically unstable.

**Table 1:** Adsorption energies (in eV) of a Pt single atom adsorbed in the most stable structure on clean and peroxide‐covered CeO_2_ surfaces as a function of the peroxide coverage (1 ML corresponds to one peroxo group per (1×1) surface unit cell). At 0.5 ML coverage for the (2×2) slabs used in the calculations, two peroxo groups allow for two non‐equivalent geometric arrangements: 1) along the unit vectors (labelled “r”=row‐wise); and 2) diagonal to the unit vectors (“d”=diagonal). The energies are referenced to the corresponding ceria surfaces and Pt bulk. Negative values indicate exothermic adsorption and unfavourable Pt aggregation (Pt−Pt bond formation).

Θ_peroxide_ (ML)						
Surface	0.0	0.25	0.5‐d	0.5‐r	0.75	1.0
(111)	2.80	1.28	1.98	1.75	1.62	0.59
(110)	0.60	−0.17	0.75	−0.08	0.11	0.12
(100)	0.31	0.14	0.46	0.30	0.56	0.64
(100)‐4O	−0.85	−0.59	−0.95	−0.24	−0.63	1.76

Next, we investigated the interaction of Pt single atoms with ceria NPs on the CeO_2_(111) surface. To this end, we constructed a model system consisting of a ceria cluster anchored on an extended CeO_2_(111)‐(7×7) slab. The particle size was adapted to that measured experimentally (i.e., about 1 nm, see Figure 2), and the entire structure was optimized by DFT accordingly (see Figure 5a, center). Obviously, new adsorption sites for Pt atoms become available at the boundary between the NP and the surrounding (111) terrace. The computed adsorption energy of a Pt single atom *E*_ads_(Pt) on these sites (0.57 eV) is substantially smaller than on clean and peroxo‐covered (111) surfaces (2.80 and 1.3–1.6 eV, respectively, Table 1). However, the (100)−4O sites exposed at the vertices of the ceria NPs present the thermodynamically most favorable adsorption sites for Pt (*E*_ads_(Pt)=−1.76 eV). We also note that depending on their location on the ceria NP the coordination environments for adsorbed Pt atoms are chemically different from those of their symmetrically equivalent counterparts on the extended ceria surfaces. In particular, at (100)−4O sites, the difference is substantial and amounts to 0.9 eV (i.e., −1.76 vs −0.85 eV, on NP vertices and the corresponding extended surface, respectively).

**Figure 4:**
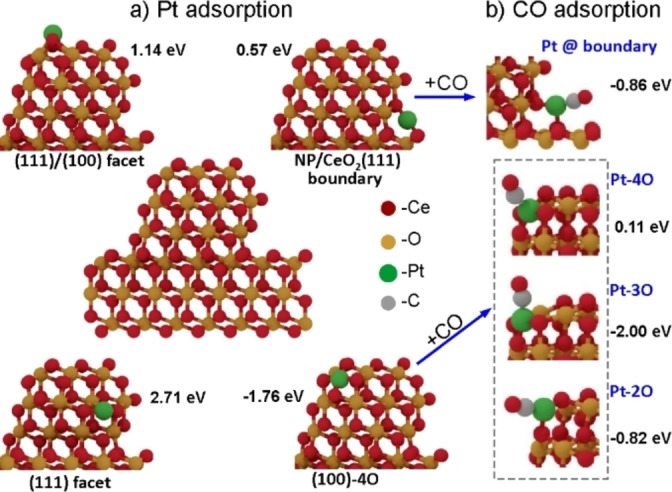
a) Adsorption of Pt atom at several different sites on a ceria NP/film composite system (Ce_178_O_356_, shown in the middle). The adsorption energies (in eV; referenced to bulk Pt) are shown adjacent to the structures. b) Optimized geometries for CO adsorption on Pt single atoms at the NP/boundary and at sites derived from Pt in a (100)−4O “nanopocket”. By removing one or two ligand oxygen atoms from the latter coordination site, other, more stable adsorption geometries for CO can be formed (termed “Pt‐3O” and “Pt‐2O”). CO adsorption energies (in eV) are indicated. (All the computed structures can be retrieved at DOI: 10.19061/iochem‐bd‐1‐181)

We further investigated the NP/terrace boundary and the square‐planar “nanopocket” Pt‐binding sites of this system for CO adsorption (Figure 4b, Figure S13). We found that CO does not adsorb on Pt in the (100)−4O site (*E*_ads_(CO)=0.11 eV), while CO adsorption is exothermic for Pt at the NP/CeO_2_(111) boundary (*E*_ads_(CO)=−0.86 eV). However, on this site the CO molecule is oriented almost parallel to the metal substrate surface and will be invisible in IRAS due the above‐mentioned selection rules. Both these findings may explain the very low intensity of the IRAS bands on the “as deposited” Pt/CeO_2_‐plasma surface as compared to that observed for the Pt/CeO_2_(111) system (Figure 3). As for the latter, the calculations performed for a Pt ad‐atom on the pristine CeO_2_(111) surface yielded *E*_ads_(CO)=−2.14 eV and a CO frequency of 2093 cm^−1^, which matches the 2087 cm^−1^ band observed on the “as deposited” Pt/CeO_2_(111) sample (Figure 3). The CO binding energy increases to −2.33 and −2.62 eV for Pt adsorbed onto 0.25 and 0.5 ML peroxo‐covered CeO_2_(111) surfaces, and the CO frequency shifts to 2081 and 2080 cm^−1^, respectively. Therefore, we can tentatively assign the IRAS band at 2104 cm^−1^ observed on the “as deposited” Pt/CeO_2_‐plasma surface to Pt single ad‐atoms adsorbed on the bare and/or peroxo‐covered CeO_2_(111) surface. Upon UHV annealing at 500 K, these atoms probably migrate to the more strongly bound boundary and nanopocket sites where they only weakly adsorb CO that leads to a considerable reduction of the signal intensity as shown in Figure 3b.

In an attempt to identify strongly bonded CO molecules remaining on the Pt/CeO_2_‐plasma surface after reaction, we evaluated several structures derived from the Pt/nanopocket site by sequential removal of O atoms from the Pt‐4O coordination as a result of their reaction with CO.^[21]^ The resulting configurations, denoted Pt‐3O and Pt‐2O in Figure 4b, revealed exothermic CO adsorption with energies amounting to −2.00 eV and −0.82 eV, respectively. As such, the Pt‐3O structure appears as likely candidate responsible for the CO IRAS band observed on the post‐reacted Pt/CeO_2_‐plasma samples.

Now we are in position to rationalize the overall impact of the plasma treatment on the structure of the Pt/CeO_2_(111) surface as illustrated in the following Scheme.

**Scheme 1:**
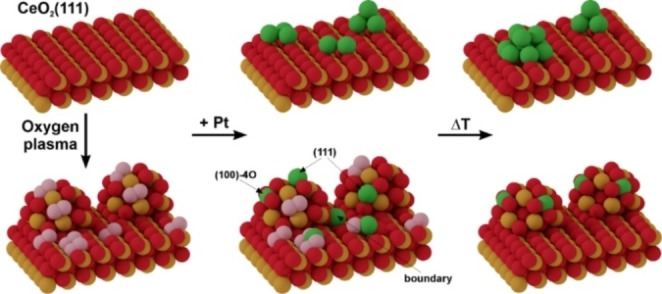
Schematic representation of the interaction of Pt atoms with pristine and oxygen plasma‐treated CeO_2_(111) films. Upon deposition on a stoichiometric CeO_2_(111) surface, Pt forms small clusters which aggregate into larger Pt NPs at elevated temperatures. Plasma pre‐treatment of the CeO_2_ surface produces peroxo species and induces surface restructuring, resulting in small ceria NPs, which act as anchoring sites either directly upon Pt adsorption or through surface migration of peroxo‐stabilized Pt single atoms. Color code: Ce: gold; O: red; Pt: green; and peroxo O_2_^2−^: pink.

It leads to two crucial modifications of the ceria surface: 1) morphological, as evident from the pronounced nanostructuring resulting in the formation of ceria nanoparticles; and 2) chemical, via surface peroxide formation. During Pt deposition, the metal atoms may either directly stick to the ceria NPs or adsorb onto the peroxo‐covered CeO_2_(111) surface in‐between, where they form isolated strongly bound complexes with the surface peroxide groups. This is in contrast to the pristine ceria film surface, where Pt atoms readily diffuse on the surface and ultimately agglomerate into Pt NPs. With increasing temperature, the peroxo‐trapped Pt atoms on the (111) terraces may further migrate to the numerous ceria NPs around and occupy sites at the particle/terrace boundary and the nanopockets. Thus, the peroxide‐covered (111) surface, firstly, prevents Pt aggregation during deposition and, secondly, acts as a reservoir for Pt single atoms on ceria NPs. Consequently, the morphological and chemical modifications of the ceria surface, as well as their synergistic interplay are critical to achieve a high density of stable Pt single atoms.

**Figure 5:**
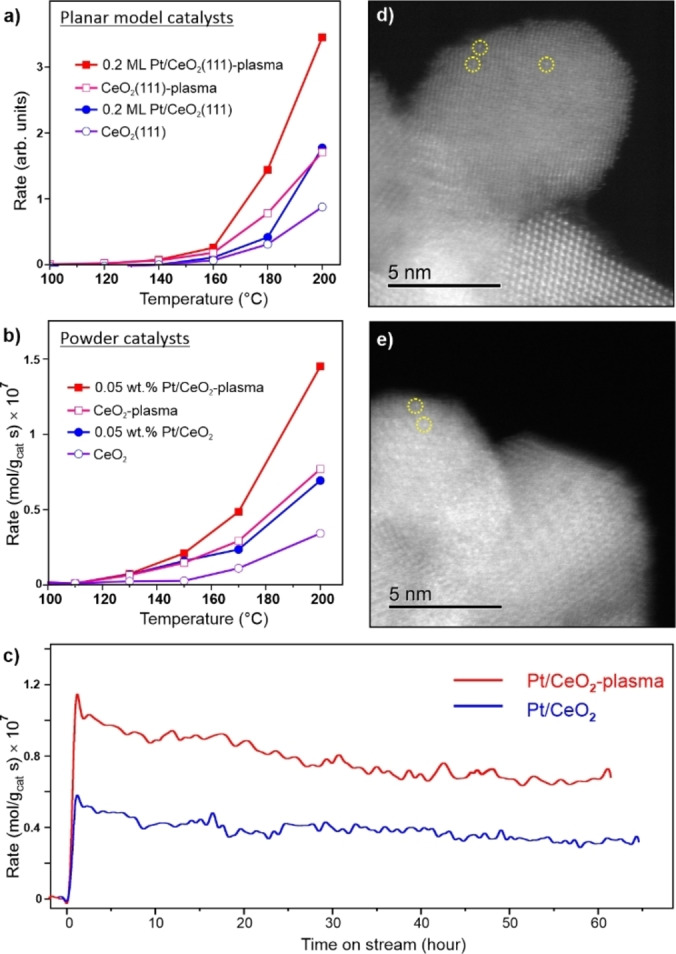
a, b) CO oxidation rate on model planar (a) and powder (b) Pt/CeO_2_ catalysts measured in reaction mixtures containing 1 % CO+5 % O_2_ and 1 % CO+20 % O_2_, respectively, (He balanced to 1 bar) during a stepwise increase of the sample temperature. The results for Pt‐free CeO_2_ supports are shown for comparison. c) Long‐term catalytic tests of “as prepared” and plasma‐treated 0.05 % Pt/CeO_2_ catalysts at 200 °C. Time zero corresponds to the start of heating to 200 °C with the rate of 10 °C min^−1^. d, e) Aberration‐corrected STEM images of 0.05 wt.% Pt/CeO_2_‐plasma catalyst before (d) and after (e) reaction at 200 °C. Some of the Pt single atoms are highlighted by circles.

The formation of highly stable Pt single atoms on the plasma pre‐treated surface shows a beneficial effect for the CO oxidation reaction. To illustrate this, Figure 5a compares steady state CO_2_ production rates measured on several planar model catalysts in a high‐pressure cell filled with 10 mbar of CO and 50 mbar of O_2_ balanced by He to 1 bar at different sample temperatures that are increased stepwise. Among the samples studied, the Pt/CeO_2_‐plasma one showed a considerably higher rate than that of Pt/CeO_2_(111). It is also interesting that the CeO_2_‐plasma film itself exhibited a higher activity as compared to the pristine CeO_2_(111) film.

To link the above‐presented model studies with real catalytic systems and to demonstrate the beneficial effect of the plasma treatment also for powder catalysts, Figure 5b shows CO_2_ production rates measured on pristine and plasma‐treated CeO_2_ and Pt/CeO_2_ powder samples (see Experimental details in the Supporting Information). A low Pt loading (0.05 wt.%) was only used in order to compare our catalysts to “conventional” Pt/CeO_2_ SACs reported in ref.^[25]^ The catalytic performance of the powder catalysts revealed basically the same trend as for the planar systems (Figure 5a). The plasma‐treated ceria exhibits higher activity than its pristine counterpart. Plasma treatment of the Pt/CeO_2_ catalyst enhances the reaction rate even further, and to a higher extent than that of observed for pure ceria, thus indicating a synergetic effect between Pt and a functionalized ceria support. The higher activity of the O_2_‐plasma treated catalysts was further confirmed by the long‐term catalytic stability tests shown in Figure 5c. These data demonstrate that the beneficial effect of the plasma pre‐treatment is long‐lasting.

On our powder samples, it was possible to directly address the peroxo formation using Raman spectroscopy.^[23b]^ Raman spectra provided strong evidence for the enhanced formation and high stability of peroxo species on the plasma‐treated samples via a characteristic Raman shift at around 835 cm^−1^ (Figure S14). Finally, we made use of aberration‐corrected scanning transmission electron microscopy (STEM), which imaged Pt single atoms on plasma‐treated Pt/CeO_2_ catalysts both before and after reaction (Figure 5d, e). The STEM data demonstrate a high stability of the plasma‐treated powder Pt SACs, in full agreement with the results obtained on the planar model systems. Although the promotional effect could further be optimized by tuning the plasma exposure conditions, our findings provide a strong basis for using plasma treatments in the rational design and stabilization of single atom catalysts.

## Conclusion

In this work, we showed that an oxygen plasma treatment of crystalline CeO_2_(111) films stabilizes Pt^2+^ single atoms which become resistant towards sintering in the CO oxidation reaction at near atmospheric pressures. We attribute the effect to: i) the fine roughening (“nano‐roughening”) of the ceria surface; and ii) the formation of peroxo species, which favor the strong interaction with Pt ultimately resulting in a uniform distribution of isolated Pt atoms and an increased single atom capacity. Such a “single‐pot” fabrication of single atom coated oxide supports, which combines both surface restructuring and active oxygen enrichment is a promising approach for the rational design of new catalysts employing single atoms.

## Conflict of interest

The authors declare no conflict of interest.

## Supporting information

As a service to our authors and readers, this journal provides supporting information supplied by the authors. Such materials are peer reviewed and may be re‐organized for online delivery, but are not copy‐edited or typeset. Technical support issues arising from supporting information (other than missing files) should be addressed to the authors.

Supporting Information
