# Supplementary material for: Highly Stable and Reactive Platinum Single Atoms on Oxygen Plasma‐Functionalized CeO2 Surfaces: Nanostructuring and Peroxo Effects
Source: Angew Chem Int Ed Engl. 2022 Mar 16;61(20):e202112640. doi: 10.1002/anie.202112640 (PMC9315031; doi:10.1002/anie.202112640)
Supplement: Supplementary file 1 — Supporting Information [file ANIE-61-0-s001.pdf]

Angewandte Chemie
Eine Zeitschrift der Gesellschaft Deutscher Chemiker

Supporting Information

Highly Stable and Reactive Platinum Single Atoms on Oxygen Plasma-Functionalized CeO_2 Surfaces: Nanostructuring and Peroxo Effects

W. Wan, J. Geiger, N. Berdunov, M. Lopez Luna, S. W. Chee, N. Daelman, N. López, S. Shaikhutdinov*, B. Roldan Cuenya**

Supporting Information

Experimental methods

The experiments were performed in an ultrahigh vacuum (UHV) chamber equipped with low energy electron diffraction (LEED), x-ray photoelectron spectroscopy (XPS), and scanning tunneling microscopy (STM), all from SPECS. The stoichiometric well-ordered $CeO_2(111)$ films were grown on a Ru(0001) single crystal as described elsewhere.[1] The crystal (9 mm in diameter, 1.5 mm in thickness, from MaTeck GmbH) was mounted onto a stainless steel sample holder having a hole of 9 mm in diameter for heating the sample from the backside using an electron beam from a W filament. A type K thermocouple was spot-welded to the edge of the crystal. Ce was vapor-deposited using an electron beam assisted evaporator (Focus EMT3) from a Mo crucible filled with Ce (99.9%, Sigma Aldrich). The thickness of the prepared $CeO_2(111)$ films was about 5 nm as determined from the attenuation of the Ru 3d signal in XPS spectra.

The reduced $CeO_{2-x}(111)$ surface (henceforth referred to as "CeO_2-red") was prepared by UHV annealing of the CeO_2 films at 1200 K for 5 min. The $CeO_2(111)$ surface enriched with monoatomic steps (denoted as "CeO_2-step") was prepared following the procedure describe in detail in Ref.[2] The oxygen plasma treatment of the $CeO_2(111)$ films was carried out with a commercial plasma source (OSPrey, from Oxford Scientific) operated in 7×10^{-6} mbar of O_2 at an anode voltage of 1 kV and an emission current of 0.5 µA. The latter samples are denoted as "CeO_2-plasma".

Pt was deposited onto ceria films using an electron beam-assisted evaporator from a Pt rod. In order to minimize metal aggregation during the deposition at room temperature, Pt was deliberately deposited at low metal flux, *i.e.*, 0.03 ML/min, as determined by a quartz microbalance (McVac), where 1 ML corresponds to one Pt atom per $CeO_2(111)$ surface unit cell, *i.e.*, 8×10^{14} cm^{-2}. During the deposition, the sample was biased at the same potential as the Pt rod. In addition, the amount of Pt was controlled by XPS through analysis of the Pt 4f signal intensity.

XPS spectra were measured with a Phoibos 150 analyzer (Specs) using an Al K_α x-ray source ($h\nu$ = 1486.6 eV). Pt 4f, Ce 3d, and O 1s core level spectra were recorded at pass energies of 25, 50, and 50 eV, respectively. Spectral analysis (background subtraction and deconvolution) was performed with the CasaXPS software.

The reactivity measurements were performed in a high-pressure cell (SPECS HPC-20) connected to the main UHV analysis chamber. The sample was heated by a halogen lamp through a quartz window. The reaction mixture consisted of 10 mbar of CO, 50 mbar of O_2 and balanced by

He to 1 bar. The gas composition in the reactor was analyzed by gas leaking through a quartz micro-capillary into a quadrupole mass spectrometer (QMS, MKS Instruments). Starting from the room temperature, the sample temperature was increased stepwise with a 50°C increment, and the CO_2 production was monitored by QMS. In order to test the stability of the Pt deposits in the CO oxidation reaction, the sample was heated to 250°C with a rate of 1 °C/s, and kept under reaction conditions for 10 min. The sample was then transferred into the analysis chamber for the XPS measurements after it was cooled down to room temperature, and the gas was pumped out.

The infrared reflection-absorption spectroscopy (IRAS) measurements were performed with a Bruker 66*ivs* FTIR spectrometer in another UHV chamber equipped with XPS (SPECS), LEED (SPECS), Ce and Pt evaporators (EFM3), plasma source (Oxford Scientific), and a home-built "high-pressure" cell (reaction volume about 1 l) for exposure to the CO oxidation reaction mixture. In this setup, the Ru(0001) crystal was spot-welded to the Ta wire for resistive heating, and the thermocouple was spot-welded to the backside of the crystal.

The ceria powder (surface area 50 m^2/g) was purchased from US Research Nanomaterials, Inc. Tetraammineplatinum(II) nitrate (TAPN) (99.995%, Sigma Aldrich) was used as the Pt precursor. The ammonium hydroxide (NH_4OH, 30% solution in water) was supplied by Acros Organics. To synthesize the 0.05 wt.% Pt/CeO_2 catalysts, 1 g of CeO_2 was crushed, sieved to a powder finer than 75 µm, and then added to a solution of deionized water mixed with NH_4OH at pH 9. Then 10 mg of TAPN were dissolved in 5 ml of deionized water, and 300 µl of this solution was added to 25 ml of an aqueous NH_4OH solution at pH 9. The resulting TAPN and NH_4OH mixture was very slowly (for about 3.5 hours) added to the support solution. It was subsequently dried at 70°C in a rotary vacuum evaporator (at 110 rpm). Finally, the catalyst was calcined in air at 450°C for 4 hours. The calcination temperature was reached with a heating rate of 10°C min^{-1}.

The O_2-plasma treatment of the nanocrystalline powder catalysts was performed in a home-built setup consisting of a glass tube (20 cm long and 2 cm inner diameter) standing upright, a vacuum pump stage, and a radio frequency plasma generator (PVM500). The glass tube is wrapped around with Cu mesh used as one electrode. The second electrode made of a tungsten rod of 2 mm in diameter goes inside a glass tube along the axis. The frit in the bottom end of the tube allow to hold the sample powder inside while plasma gas passes through from the bottom. Typically, the glass vessel is evacuated down to ~ 30 mbar and then oxygen is introduced with a flow rate of 20 ml min^{-1} using a mass flow controller (Bronkhorst) and the plasma was ignited. The powder samples levitated within the tube, suggesting uniform exposure to the plasma. For the samples studied in the manuscript, the plasma treatment lasted for 1 hour. XPS inspection of the plasma-treated

powder samples revealed no contamination, in particular of W. Note also that analysis of the elemental composition of our catalysts by ICP-MS (ThermoScientific iCAP RQ) did not reveal any loss of Pt upon O_2-plasma treatment.

The reactivity of the powder samples in CO oxidation was tested in a tubular packed-bed flow reactor. The gas phase composition was analyzed with a quadrupole mass spectrometer (Hiden 20). The prepared catalyst was sieved to below 75 µm, physically mixed with freshly calcined silica gel at a 1:4 wt. ratio, then further diluted 5:1 by acid-purified SiO_2 and subsequently loaded into the glass tube reactor. The CO oxidation reaction rate was measured at steady state in a mixture of 1 vol.% CO and 20 vol.% O_2, (He balance). For the comparative study shown in Fig. 5b in the main text, the reaction rate was measured at several different temperatures increased stepwise from room temperature to 200°C, at least for 1 h at each temperature. For the long-term catalytic tests shown in Fig. 5c, the freshly prepared samples were purged in Ar at 200°C for 30 min and subsequently exposed to the reaction mixture after cooling to the room temperature. Then the samples were heated to 200°C with the rate of 10°C/min.

Aberration-corrected scanning transmission electron microscopy (STEM) images of powder samples were obtained with a 200 kV JEOL JEM ARM200F probe/image-corrected TEM (JEOL Ltd). The samples were dispersed in the ethanol and drop-casted onto the TEM grids.

Raman spectra were measured with an inVia™ Raman Microscope (Renishaw) using a 532 nm excitation laser. For each sample, five different spots were inspected. The acquisition time per each of the three scans in each spot was 20 s. The spectra were normalized to the principal F_{2g} peak at 465 cm^{-1}. The spectra presented are the average of the normalized spectra at all five spots.

Computational methods

Simulations were performed with the Vienna Ab initio Simulation Package (VASP, version 5.4.4),[3] employing the generalized gradient functional by Perdew, Burke and Ernzerhof (PBE)[4]. Core electrons were treated within the projector-augmented wave (PAW) method, [5] while valence electrons were expanded in plane-waves with a basis set cut-off of 500 eV. An additional Hubbard correction (DFT+U) was applied to the Ce(4f) band following Dudarev et al.[6] using an on-site, effective U-parameter of 4.5 eV.

Optimization of bulk ceria yields a theoretical lattice parameter of 5.491 Å. The (111), (110) and (100) surfaces were modelled using (2×2) slabs. Stepped surfaces were prepared accordingly as

(1×2) supercells. At least 10 Å of vacuum were added on top of the slabs. The bottom layers of the slabs (approx. half of the total number) were kept fixed at the optimized bulk positions, while the upper layers were allowed to relax. A dipole correction along the surface normal was applied throughout. Sampling of the Brillouin zone was performed with Γ-centred k-point grids with a reciprocal grid spacing of about 0.025 $Å^{-1}$. Convergence of atomic positions was assumed when the absolute forces acting on each atom fell below 0.15 eV/Å. Spin polarization was accounted for when necessary. Peroxide formation energies and adsorption energies of Pt and CO were obtained by step-wise structural relaxations, starting from the pre-optimized pristine surfaces. For the ceria NP/slab composite model, we employed an initial octahedral nanoparticle, following the well-known Wulff construction and applied several cuts, ensuring CeO_2 stoichiometry. The resulting ceria nanoisland was anchored on a CeO_2(111)-(7×7) slab of nine atomic layers. The full system consists of 178 cerium and 356 oxygen atoms (film - $Ce_{147}O_{294}$; NP - $Ce_{31}O_{62}$) and has a total expansion of 27.18 × 27.18 × 7.94 $Å^3$. Due to the large cell dimensions, optimizations were carried out at Gamma-point.

Evaluation of CO vibrational frequencies was done by applying small, step-wise displacements of the relevant platinum, carbon and oxygen atoms (VASP-tag NFREE=2). Due to the high computational cost, a modified version of the NP/slab composite system, only keeping the surface layer of the underlying slab, was used and the Pt was kept fixed. Reference calculations have shown that deviations incurred by this approximation stay below 10 cm^{-1}, validating the approach. Additionally, in the case of the extended low-index surfaces, higher precision was applied by setting the VASP-tag PREC to Accurate. Lastly, all frequencies were scaled by referencing with the theoretical and experimental value of molecular CO (see Table S12).

The plasma was modelled as isolated oxygen atoms (radicals). Binding energies of the O 1s level at surface were calculated within the final-state approximation[7] and referenced to the O 1s signal of oxygen in a ceria bulk (2×2×2) supercell.

Results

The complex Ce 3d spectra in CeO_2 are commonly rationalized in terms of three spin orbital pairs of the $Ce3d^94f^0O2p^6$, $Ce3d^94f^1O2p^5$ and $Ce3d^94f^2O2p^4$ final states and referred to as 4f', 4f'' and 4f''', respectively in Figure S1. [1b] The Ce^{3+} ions, if present, manifest itself by additional signals, corresponding to the 4f'' and 4f''' final states, which overlap with those of Ce^{4+}. As a result, the amount of Ce^{3+} can only be determined by a relatively complex deconvolution procedure.[1b]

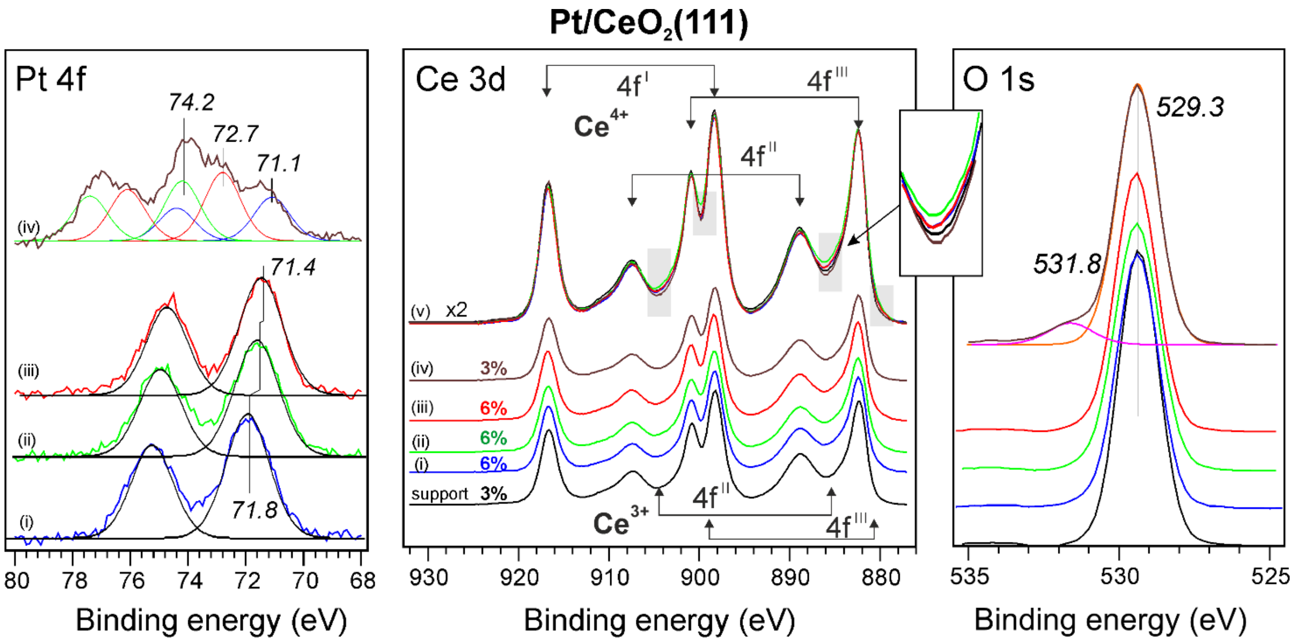

Figure S1. Pt 4f, Ce 3d, and O 1s spectra of the $Pt/CeO_2(111)$ sample: (i) as deposited at 300 K; (ii) after thermal flash to 523 K; (iii) after annealing at 523 K for 5 min; (iv) after the CO oxidation reaction. (v) Direct comparison of the Ce 3d spectra by normalizing to the intensity of the peak at 882 eV. The regions of spectral changes are highlighted by gray boxes. The relative amount of Ce^{3+} is indicated adjacent to the Ce 3d spectra. The O 1s signal at 531.8 eV is assigned to carbonate species formed under reaction conditions.

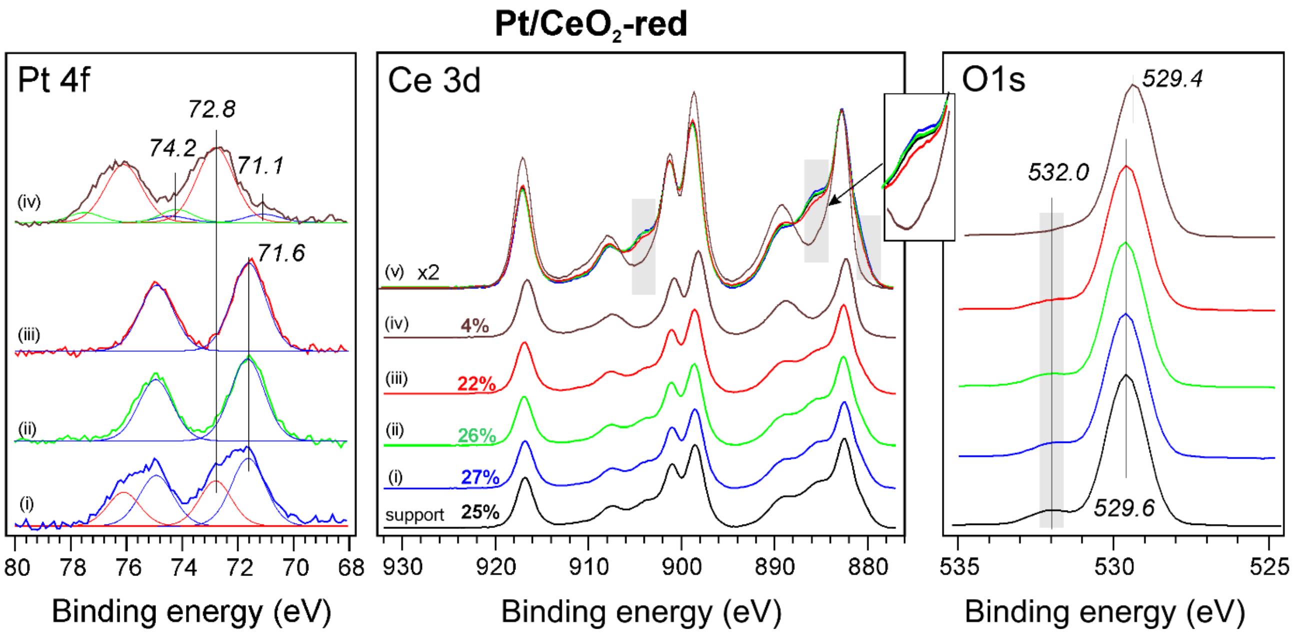

Figure S2. Pt 4f, Ce 3d and O 1s spectra of the Pt/CeO_2-red sample: (i) as deposited at 300 K; (ii) after thermal flash to 523 K; (iii) after annealing at 523 K for 5 min; (iv) after the CO oxidation reaction. (v) Direct comparison of the Ce 3d spectra by normalizing to the intensity of the peak at 882 eV. The regions of spectral changes are highlighted by gray boxes. The relative amount of Ce^{3+} is indicated adjacent to the Ce 3d spectra. The O 1s signal at 532 eV in the "as prepared" sample is tentatively assigned to hydroxyl species formed by reaction with the residual gases in the UHV background.

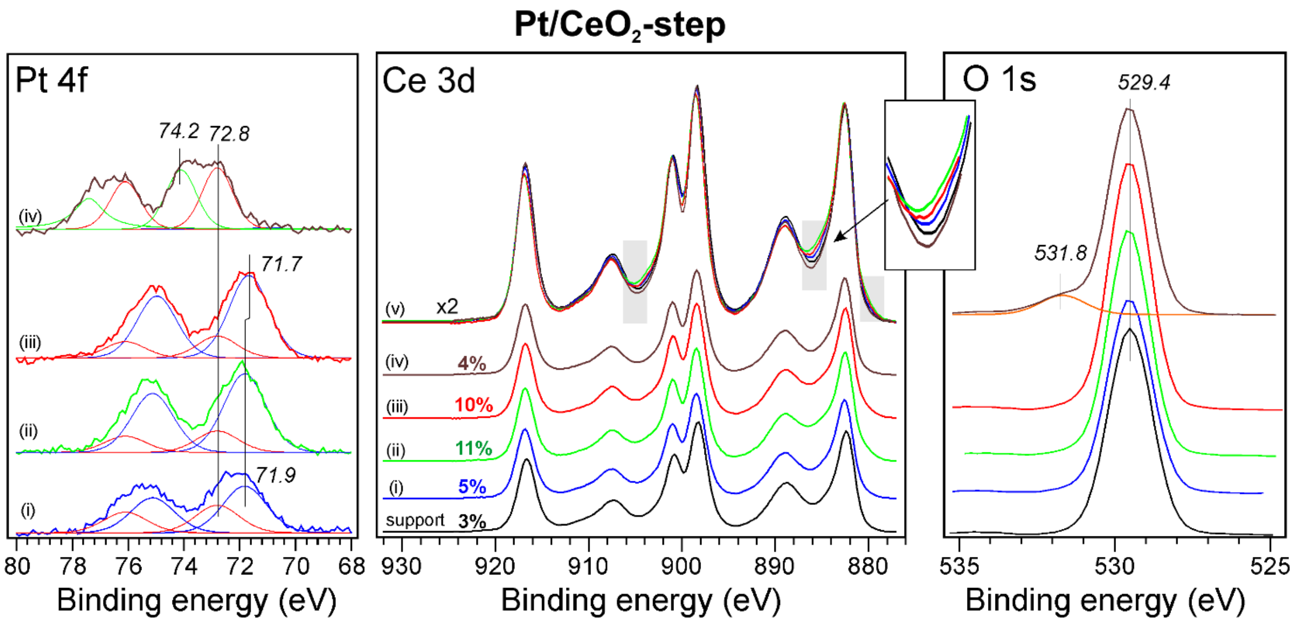

Figure S3. Pt 4f, Ce 3d and O 1s spectra of the Pt/CeO_2-step sample: (i) as deposited at 300 K; (ii) after thermal flash to 523 K; (iii) after annealing at 523 K for 5 min; (iv) after the CO oxidation reaction. (v) Direct comparison of the Ce 3d spectra by normalizing to the intensity of the peak at 882 eV. The spectral changes are highlighted by gray boxes. The relative amount of Ce^{3+} is indicated adjacent to the Ce 3d spectra. The O 1s signal at 531.8 eV is assigned to carbonate species formed under reaction conditions.

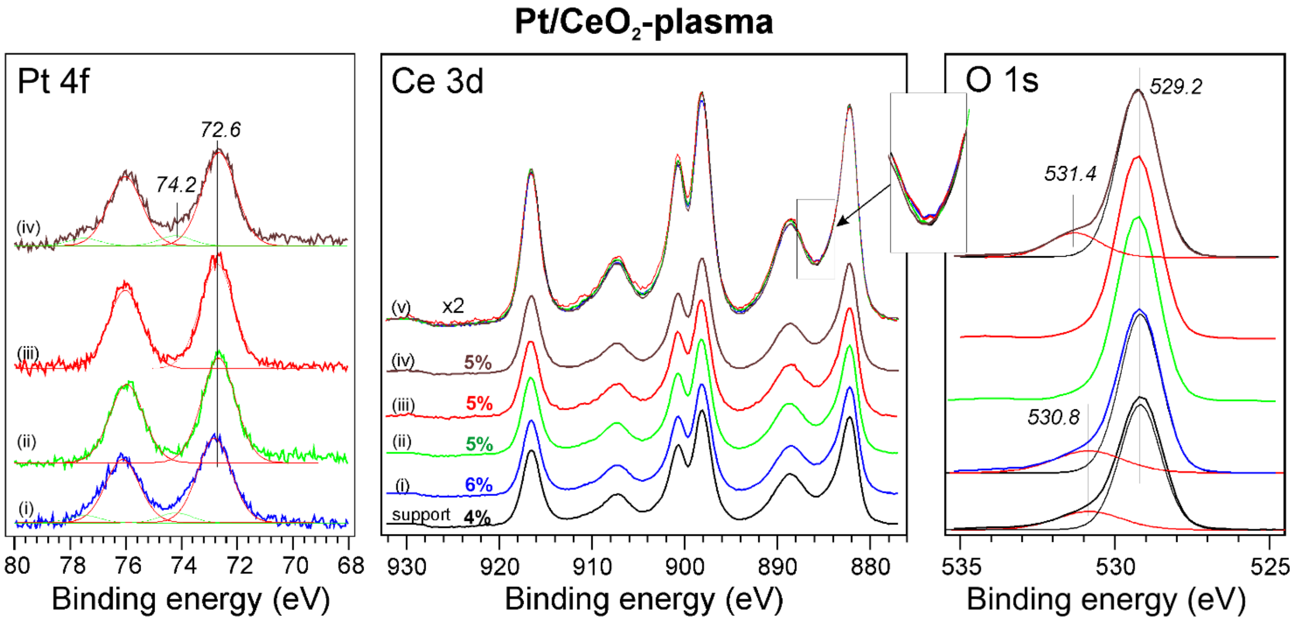

Figure S4. Pt 4f, Ce 3d and O 1s spectra of the Pt/CeO_2-plasma sample. (i) as deposited at 300 K; (ii) after thermal flash to 523 K; (iii) after annealing at 523 K for 5 min; (iv) after the CO oxidation reaction. (v) Direct comparison of the Ce 3d spectra by normalizing to the intensity of the peak at 882 eV. The relative amount of Ce^{3+} is indicated adjacent to the Ce 3d spectra. The O 1s signal at 530.8 eV is assigned to peroxo species (see the main text) which overlaps with carbonates-related signals formed under reaction conditions.

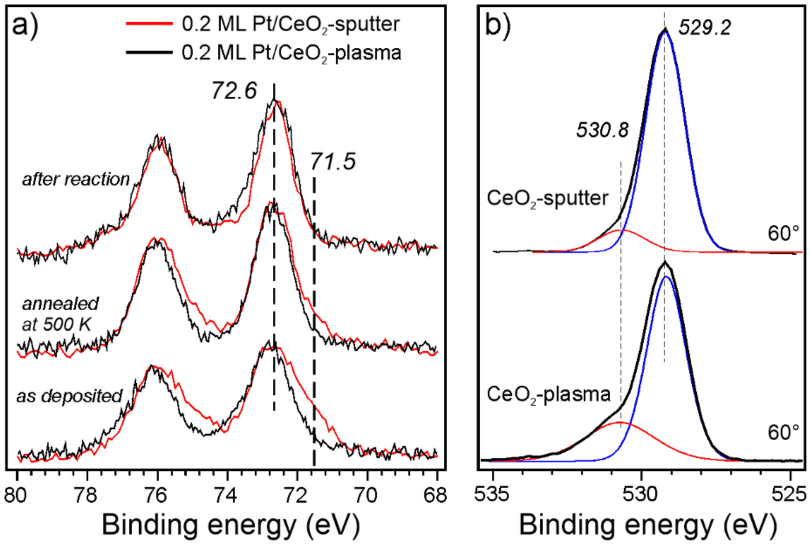

Figure S5. Comparison of Pt 4f (a) and O1 s (b) spectra of a plasma-treated CeO_2(111) film and a surface prepared by Ar^+-ion sputtering (500 eV, 5 min) and subsequent oxidation in 10^{-6} mbar O_2 at 500 K.

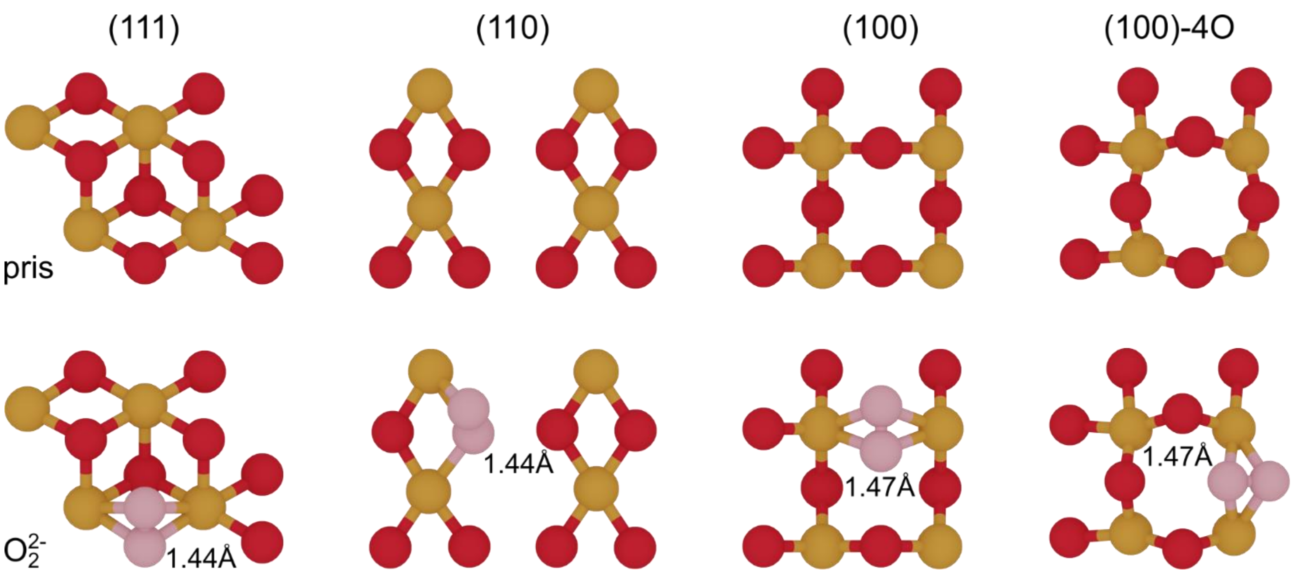

Figure S6. Top views of the pristine low-index surfaces and the structures of peroxide species formed by the reaction with oxygen radicals. As annotated, the peroxide groups can be identified by their oxygen-oxygen bond length of about 1.4 Å. The (100) surface can well accommodate the second oxygen atom within its surface layer, while a bridging position is adopted on (111) and it is considerably protruding outwards for (110). Color code: Ce - gold, O - red, O_2^{2-} - pink.

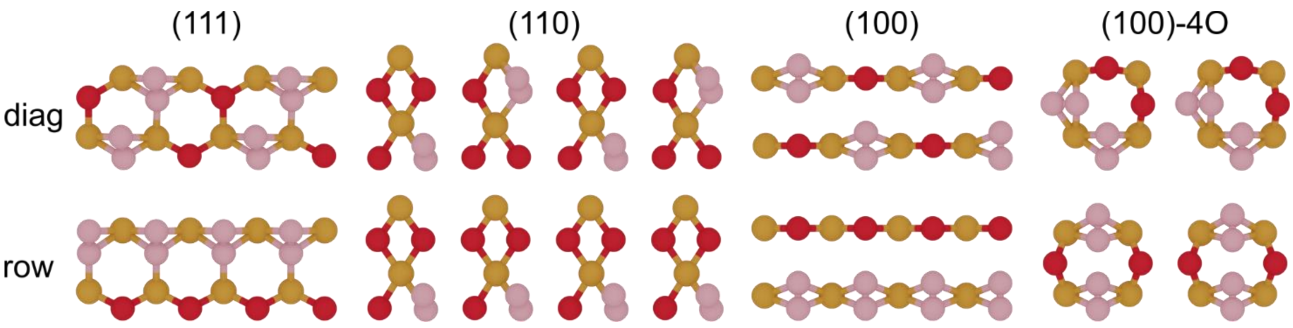

Figure S7. Top views of the different relative arrangements (diagonal vs. row-wise) of low-index surfaces with 0.5 ML peroxide coverage that arise on the considered (2×2) super cells. For better visualisation only the surface layers of extended (4×2) supercells are depicted here. Color code: Ce - gold, O - red, O_2^{2-} - pink.

Table S1. PBE+U core-level binding energies (in eV) for oxygen species on low-index ceria surfaces at varying peroxide coverages. The presented values correspond to the average results, as in each case, all of the oxygen atoms located in the surface layer were sampled. The values are always referenced to the simulated O 1s signal of an oxygen atom in a ceria bulk (2×2×2) supercell. For 0.5 ML, two different geometric arrangements of the peroxide groups are possible (denoted as "diag" and "row", see Figure **S8**).

		$\Theta_{peroxide}$ [ML]					
**Surface**	**Species**	**0.0**	**0.25**	**0.5-diag**	**0.5-row**	**0.75**	**1.0**
**(111)**	O^{2-}	-0.81	-0.85	-0.84	-0.85	-0.82	-
	O_2^{2-}	-	1.59	1.67	1.64	1.68	1.70
**(110)**	O^{2-}	-0.86	-0.80	-0.78	-0.78	-0.74	-0.66
	O_2^{2-}	-	1.19	1.37	1.15	1.33	1.37
**(100)**	O^{2-}	-1.14	-1.23	-1.39	-1.26	-1.19	-
	O_2^{2-}	-	1.52	1.47	1.43	1.39	1.19

Table S2. Geometric properties of the ceria low-index surfaces. The values were obtained for supercells extending two unit cells along x and y. The tabulated properties are the total surface area, $\mathbf{A_{surf}}$, the exposed surface area per cerium atom, $\mathbf{A_{surf}/Ce}$ (each slab exposes four cerium atoms), the surface energy, **γ,** in units of J/m^2 and eV/Å^2, and the relaxation energy, $\mathbf{E_{relax}}$, which is obtained as the energy difference between the energy of the optimized and unrelaxed slab (created from ceria bulk).

**Surface**	$\mathbf{A_{surf}}$ [Å^2]	$\mathbf{A_{surf}}$ **/ Ce** [Å^2]	**γ** [J/m^2]	**γ** [eV/Å^2]	$\mathbf{E_{relax}}$ [eV]
**(111)**	52.228	13.057	0.698	0.044	-0.011
**(110)**	85.288	21.322	1.061	0.066	-1.298
**(100)**	60.308	15.077	1.462	0.091	-2.446

Table S3. Peroxide formation energies for the low-index surfaces of ceria at different coverages, (coverages given relative to the number of surface cerium atoms). All values are in units of eV and are referenced to the energy of the pristine surface and the total energy of the adsorbed oxygen radicals: $\Delta E = E_{total} - E_{pris} - N_O E_O$. The values in parenthesis denote relative changes of the peroxide formation energies for incremental increases in coverage. For 0.5 ML, two different geometric arrangements of the peroxide groups are possible (denoted as "diag" and "row", see Figure **S8**).

	$\Theta_{peroxide}$ [ML]				
**Surface**	**0.25**	**0.5-diag**	**0.5-row**	**0.75**	**1.0**
**(111)**	-2.675 (-2.675)	-5.223 (-2.547)	-5.331 (-2.656)	-7.731 (-2.400)	-10.093 (-2.362)
**(110)**	-2.872 (-2.872)	-5.680 (-2.809)	-5.720 (-2.849)	-8.348 (-2.628)	-11.001 (-2.653)
**(100)**	-3.362 (-3.362)	-6.732 (-3.370)	-6.559 (-3.197)	-9.691 (-2.959)	-12.669 (-2.978)

Table S4. Surface energies of the low-index ceria surfaces with various peroxide coverages in eV/Å^2 (values in J/m^2 are given in parentheses). As we only allow relaxation on one side of the symmetrically cleaved slabs, the values for the pristine surfaces were calculated as $\gamma = \left(E_{relax} - (E_{unrelax} + NE_{bulk})/2\right)/A$, where $\mathbf{E_{relax}}$ is the total energy of the relaxed slab, $\mathbf{E_{unrelax}}$ is the energy of the unrelaxed, symmetric slab, as built from optimized ceria bulk, **N** is the number of cerium atoms in the slab and $\mathbf{E_{bulk}}$ is the bulk energy per ceria formula unit, and **A** the surface area. To account for the presence of peroxides, the formula has to be adapted accordingly $\gamma = \left(E_{relax} - N_O E_O - (E_{unrelax} + NE_{bulk})/2\right)/A$ where $\mathbf{N_O}$ and $\mathbf{E_O}$ are the number of surface peroxide groups and the reference energy of atomic oxygen, respectively. Entropic contributions were neglected. Due to the fact that atomic oxygen is a high-energy reference and considerable stabilisation is achieved by its reaction with lattice oxygen, the values for peroxide-covered surfaces can become negative. [8]

	$\Theta_{peroxide}$ (ML)					
**Surface**	**0.0**	**0.25**	**0.5-diag**	**0.5-row**	**0.75**	**1.0**
**(111)**	0.044 (0.698)	-0.008 (-0.123)	-0.056 (-0.904)	-0.059 (-0.937)	-0.104 (-1.674)	-0.150 (-2.398)
**(110)**	0.066 (1.061)	0.033 (0.522)	0.000 (-0.006)	-0.001 (-0.013)	-0.032 (-0.507)	-0.063 (-1.005)
**(100)**	0.091 (1.462)	0.035 (0.568)	-0.020 (-0.327)	-0.018 (-0.281)	-0.069 (-1.113)	-0.119 (-1.904)

Table S5. Gibbs free energy differences for peroxide formation on the low-index ceria surfaces at different peroxide coverages. Neglecting pV-terms and configurational entropic contributions of the adsorbed oxygen radicals, the Gibbs free energies can be obtained from the DFT-calculated internal energies, **U**, via $\Delta G = \Delta U + TS^0$. To this end, we employed the standard entropy of atomic oxygen, $\mathbf{S^0}$ = 1.669×10^{-3} Jmol^{-1}K^{-1} and room temperature, **T** = 300 K.[9] All energies are given in eV and the values in parenthesis correspond to changes upon incremental increases in coverage.

	$\Theta_{peroxide}$ [ML]				
**Surface**	**0.25**	**0.5-diag**	**0.5-row**	**0.75**	**1.0**
**(111)**	-2.175 (-2.175)	-4.221 (-2.046)	-4.330 (-2.155)	-6.228 (-1.899)	-8.090 (-1.862)
**(110)**	-2.371 (-2.371)	-4.679 (-2.308)	-4.719 (-2.348)	-6.846 (-2.127)	-8.997 (-2.152)
**(100)**	-2.861 (-2.861)	-5.731 (-2.869)	-5.558 (-2.696)	-8.189 (-2.458)	-10.666 (-2.477)

Table S6. Surface energies of low-index ceria surfaces with various peroxide coverages in units of eV/Ce. This convention was chosen to facilitate usage of the values in the thermodynamic model for the CeO_x NP formation, as outlined in Note **S3**. The values for the pristine surfaces were calculated *via* $\gamma_{per\ Ce} = [(E_{unrelax} - E_{bulk})/2 + \Delta E_{relax}]/N_{Ce,\ surf}$, while in the presence of peroxides, the reaction Gibbs energy was additionally included: $\gamma'_{per\ Ce} = \gamma_{per\ Ce} + \Delta G/N_{Ce,\ surf}$.

	$\Theta_{peroxide}$ (ML)					
**Surface**	**0.0**	**0.25**	**0.5-diag**	**0.5-row**	**0.75**	**1.0**
**(111)**	0.569	0.025	-0.486	-0.514	-0.988	-1.454
**(110)**	1.413	0.820	0.243	0.233	-0.299	-0.837
**(100)**	1.375	0.660	-0.057	-0.014	-0.672	-1.291

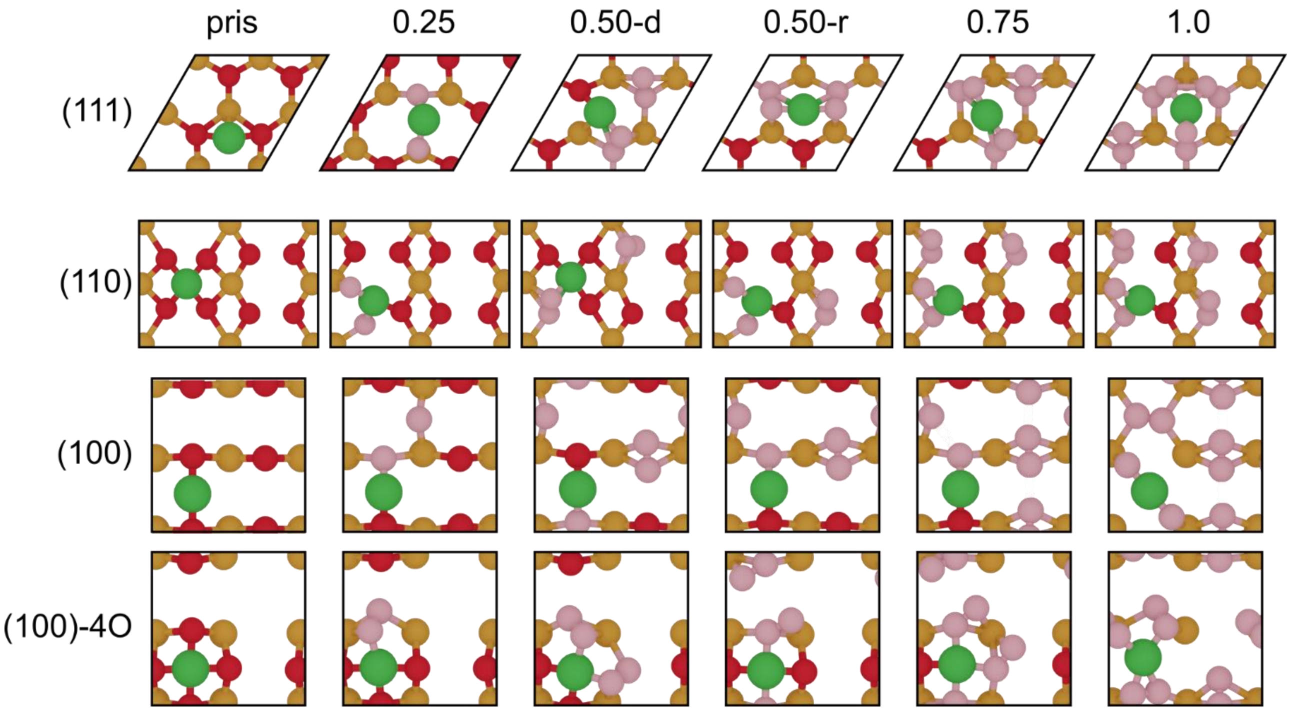

Figure S8. Top views of the energetically most favourable optimized structures of low-index ceria surfaces with different peroxide coverages and adsorbed Pt. Color code: Ce - gold, O - red, O_2^{2-} - pink, Pt - green.

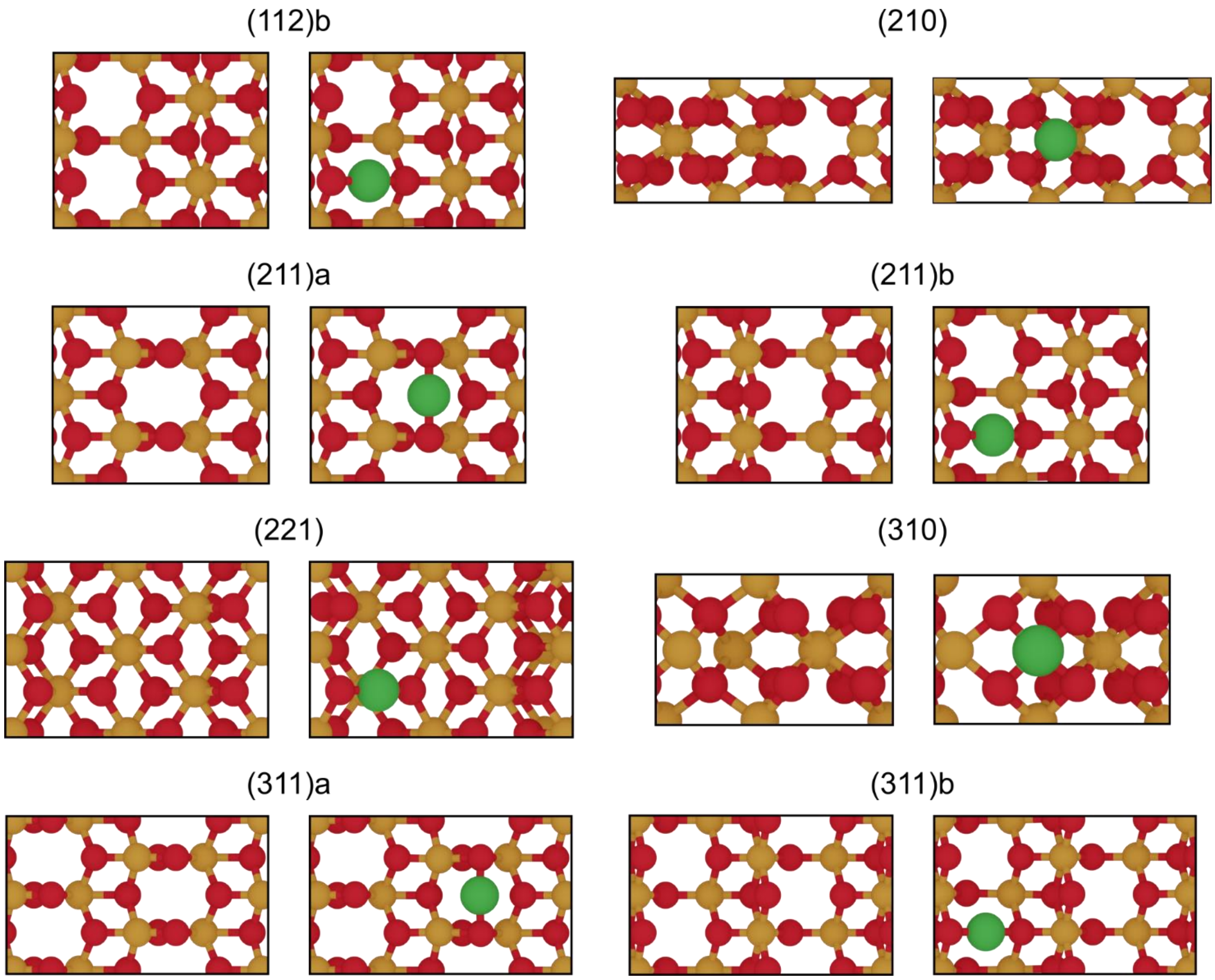

Figure S9. Top views of the full set of stepped surfaces for the approximation of the edge energy used in the thermodynamic model of ceria NP formation and the consideration of alternative binding sites for SA-Pt. Color code: Ce - gold, O - red,O_2^{2-} - pink, Pt - green.

Table S7. Geometric properties, surface energies and Pt adsorption energies for the stepped surfaces. **a** and **b** denote different oxygen localisations at the surface steps (see Figure **S13**). Platinum adsorption energies, $\mathbf{\Delta E_{ads}^{Pt}}$, are given relative to bulk metal, and peroxide formation energies, $\mathbf{\Delta E_{ads}^{O}}$, are referenced to atomic oxygen. **Pt-nO**, describes the coordination environment around the platinum atom, where **2O** corresponds to linear bonding between two lattice oxygen atoms, similar to (100), while **4O** denote a square-planar geometry involving four oxygen atoms, similar to the nanopockets on reconstructed (100).

**Surface**	$\mathbf{A_{surf}}$ [Å^2]	**γ** [J/m^2]	**γ** [eV/Å^2]	$\mathbf{\Delta E_{ads}^{Pt}}$ [eV]	**Pt-nO**	$\mathbf{\Delta E_{ads}^{O}}$ [eV]
**(112)b**	36.931	0.987	0.062	0.590	2O	-3.278
**(210)**	67.426	1.945	0.121	-3.581	4O	-5.321
**(211)a**	36.931	1.252	0.078	0.172	2O	-4.181
**(211)b**	36.931	1.315	0.082	0.054	2O	-3.281
**(221)**	45.231	0.834	0.052	1.425	2O	-2.763
**(310)**	47.677	1.807	0.113	-0.734	4O	-4.699
**(311)a**	50.004	1.346	0.084	1.111	2O	-2.784
**(311)b**	50.004	1.133	0.071	0.626	2O	-3.269

Note S1. From the consideration of an extensive set of stepped surfaces, as presented in Table **S7** and Figure **S9**, it can be concluded that binding sites which provide better stabilisation for Pt than extended (111) can be provided by these systems. Nonetheless, adsorption energies are still mostly endothermic with respect to Pt bulk. Square-planar coordination environments on (210) and (310) result in strongly exothermic Pt adsorption energies. However, due to the high amount of under-coordinated sites, these facets also entail the highest surface energies. All in all, square-planar "nanopockets" exposed on the NPs and restructured (100) still provide the best compromise between exceptional stabilisation of Pt SA and feasible surface energies.

Table S8. Platinum adsorption energies (in eV) on low-index ceria surfaces with different peroxide coverages. Only the values for the energetically most favourable structures are given. The energies are referenced to the peroxide-covered surfaces and Pt bulk (atomic Pt, in parentheses).

	$\Theta_{peroxide}$ [ML]					
**Surface**	**0.0**	**0.25**	**0.5-diag**	**0.5-row**	**0.75**	**1.0**
**(111)**	2.798 (-2.793)	1.281 (-4.310)	1.975 (-3.616)	1.753 (-3.838)	1.622 (-3.969)	0.591 (-5.000)
**(110)**	0.600 (-4.991)	-0.166 (-5.757)	0.749 (-4.842)	-0.079 (-5.671)	0.107 (-5.484)	0.119 (-5.472)
**(100)**	0.309 (-5.282)	0.138 (-5.453)	0.464 (-5.128)	0.301 (-5.290)	0.564 (-5.027)	0.642 (-4.949)
**(100)-4O**	-0.854 (-6.445)	-0.586 (-6.177)	-0.946 (-6.537)	-0.236 (-5.827)	-0.634 (-6.225)	1.758 (-3.833)

Note S2. From the structures presented in Figure **S8** and the values listed in Table **S8** it can be concluded that the presence of peroxide groups considerably increases the adsorption of Pt on the extended (111) surfaces. While adsorption of a Pt atom might lead to the breaking of a peroxo group (e.g. on (111)-0.25 ML), resulting in a more exothermic reaction energy, this effect seems not sufficient to stabilize a Pt single atom under reaction conditions. Square-planar "nanopockets" on restructured (100) (denoted as "(100)-4O") present the most favourable binding site. Therefore, the presence of peroxides alone does not constitute the decisive factor for achieving a high-density of stable SA-Pt for the catalyst in its resting state. However, they can lead to the initial trapping of Pt atoms upon deposition and prevent metal agglomeration.

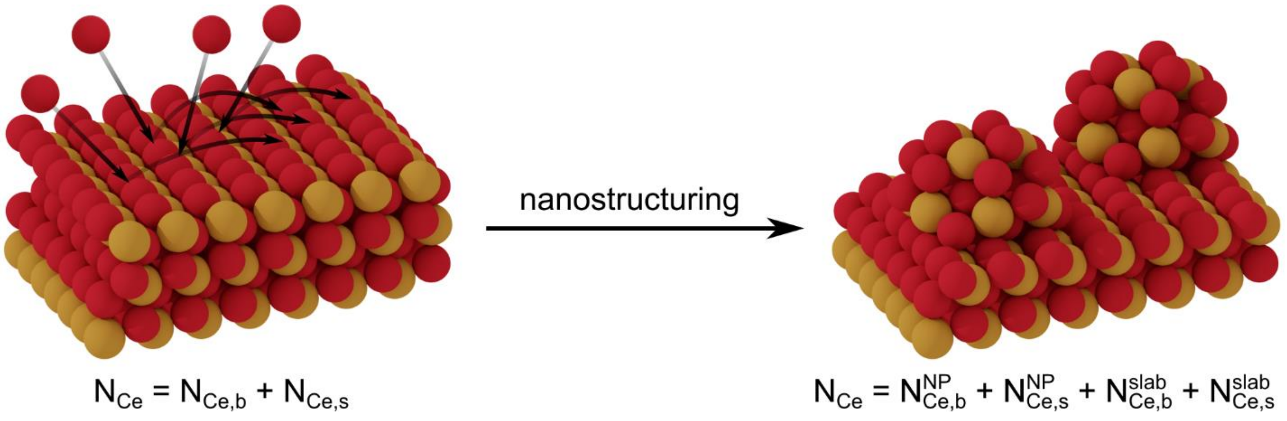

Figure S10. Schematic visualisation of the restructuring of the ceria thin film that results in the formation of CeO_2 NPs with simultaneous reduction of the film thickness. Color code: Ce - gold, O - red.

Note S3. In the following, the derivation of our thermodynamic model on the process of nano-structuring of the ceria thin film through the oxygen plasma treatment is outlined. It should be noted that the objective of this thermodynamic model is not to determine the particle size, but rather to highlight the fact that the observed nanostructuring of the initially flat ceria film is an energetically feasible process.

As the material forming the CeO_2 NPs must originate from the initial film, the overall amount of ceria is considered to remain constant, while the underlying slab in turn becomes thinner. This is schematically shown in Figure **S6**. Furthermore, thermodynamic equilibrium between both systems is assumed:

$$E_b + E_s = E_b^{'} + E_s^{'} + E_b^{NP} + E_s^{NP} + E_{edge}^{NP} + E_{vert}^{NP} \tag{1}$$

Here, **E** are the internal energies of the bulk (*b*) and surface (*s*) of the CeO_2 nanoparticle (*NP*), the initial, and the modified thin film (after restructuring, *primed*). Additionally, we account for energy contributions by edge and vertex (*vert*) species that are necessarily exposed at the real ceria NPs. The terms in Eq. 1 can be written as:

$$N_b E_f + N_s(E_f + \gamma) = N_b^{'} E_f + N_s^{'}(E_f + \gamma) + N_b^{NP} E_f + N_s^{NP}(E_f + \bar{\gamma}) + E_{edge}^{NP} + E_{vert}^{NP} \tag{2}$$

,with **N** representing the number of cerium atoms (and therefore CeO_2 formula units) in each respective structure, $\mathbf{E_f}$ the ceria bulk formation energy and $\boldsymbol{\gamma}$ the surface energy of the (111) surface at 0.5 ML peroxide coverage. Since additional facets than (111) are exposed on the CeO_x NPs, we employ a modified, effective surface energy, $\bar{\boldsymbol{\gamma}}$. Enforcing the conservation of ceria formula units entails:

$$N_b + N_s = N_b^{'} + N_s^{'} + N_b^{NP} + N_s^{NP} \tag{3}$$

and the bulk energy contributions cancel. Thus, Eq. 2 simplifies to:

$$N_s\gamma = N_s^{'}\gamma + N_s^{NP}\overline{\gamma} + E_{edge}^{NP} + E_{vert}^{NP} \tag{4}$$

with N_s' given by the difference in the number of surface cerium atoms of the non-restructured film, N_s, and the interface area where the nanoisland is anchored, N_i:

$$N_s^{'} = N_s - N_i \tag{5}$$

Additionally, the assumption of a half-spherical CeO_x NP shape with radius **R** allows for the resolution of the total surface energy of the ceria NP:

$$N_s^{NP}\overline{\gamma} \approx 2\pi R^2\overline{\gamma} \tag{6}$$

and the edge energy contribution:

$$E_{edge}^{NP} \approx 2\pi R E_{edge} \tag{7}$$

The latter is approximated by the product of the circumference of the CeO_x NP and the edge energy per length unit, E_{edge}. For the calculation of the circular interface area between the ceria NP and the underlying film, double counting of the edge atoms that are treated separately has to be avoided. Therefore, we employ a radius (**R-2r**), given by the difference between the radius of the ceria NP, **R**, and the diameter of a Ce atom, **2r**:

$$N_s^{'}\gamma \approx \pi(R-2r)^2\gamma \tag{8}$$

It should be noted that the transformation from CeO_2 formula units to surface area in Eqs. 6 - 8 necessarily entails an appropriate unit change for the surface energies, which we treat as implied along the derivation. Lastly, we approximate the total energy contribution of vertex atoms, E_{vert}^{NP}, by the product of the energy of one such species, E_{vert}, multiplied with their quantity, N_{vert}:

$$E_{vert}^{NP} \approx N_{vert}E_{vert} \tag{9}$$

From Eqs. 4 - 9, we obtain:

$$N_s\gamma = (N_s - N_i)\gamma + 2\pi R^2\overline{\gamma} + 2\pi R E_{edge} + N_{vert}E_{vert} \tag{10}$$

,where the term $N_s\gamma$ on the left corresponds to the total surface energy of the film before restructuring, while the right side is composed by the different energy contributions occurring in the composite system. Eq. 10 can then be rewritten to:

$$0 = (2\pi\overline{\gamma} - \pi\gamma)R^2 + \left(4\pi r\gamma + 2\pi E_{edge}\right)R - 4\pi r^2\gamma + N_{vert}E_{vert} \tag{11}$$

and solved for **R**. Finally, we estimated the edge energy, $\mathbf{E_{edge}}$, through a sampling of representative low-energy stepped surfaces (Table **S10**) and derived the average energy of a vertex atom, $\mathbf{E_{vert}}$, along with the number of sites, $\mathbf{N_{vert}}$, from an exemplary ceria NP (see Figure **S12** and Table **S11**).

To obtain the effective surface energy, $\bar{\boldsymbol{\gamma}}$, of the ceria NP, we assumed equal exposure of (111) and (100) at half and full peroxide coverage (see Table **S7**), respectively. From the Gibbs free energies listed in Table **S5**, it is evident that the energy gained by peroxide formation on the (100) surface is significantly higher than on (111), even up to full coverage. Thus, areas on the NPs that expose (100) will get saturated before a significant build-up on (111) occurs. Furthermore, a high coverage on (100) and the resulting energy gain through the reaction with oxygen radicals are crucial assumptions for the model, as this presents a major part of the stabilisation that is achieved *via* restructuring. We attribute the energy difference between the two facets to the initial lower coordination of surface oxygen on (100) and to the fact that it can incorporate peroxide groups almost seamlessly into its surface layer (see Figure **S6**), which is not the case for (111). Lastly, peroxide formation on (111) with coverages higher than 0.5 ML becomes less favourable as on the pristine surface. Thus, further reaction with oxygen plasma will occur at areas that contain less peroxides, averaging out the overall coverage.

The input parameters for Eq. 11 are listed in Table **S9**. They result in a ceria NP with diameter of about 5 nm, in reasonable agreement with the experimental observation. To further test the robustness of our model with respect to these parameters, we varied their values and investigated the influence on the predicted NP radius. Variations of the effective surface energy of the ceria NP were considered through the relative fractions of the two different surfaces:

$$\bar{\gamma} = f\gamma_{111}^{0.5ML} + (1 - f)\gamma_{100}^{1.0ML} \quad (12)$$

Furthermore, the effect of the peroxide coverage on (111) (while keeping a high coverage on (100)) and variations of the edge and vertex energies were investigated. As the derivations of edge and vertex energies from the employed model systems (stepped surfaces/explicit ceria NP) are not straightforward, their effects were evaluated by *a posteriori* modifications of the values obtained from our model systems. As shown in Figure **S11**, the predicted NP diameter generally stays within reasonable boundaries for all considered variations of the input parameters, confirming the generality and applicability of our model under the given assumptions.

Table S9. Final input parameters for Eq. (11). The given values are the surface energy of (111) with 0.5 ML peroxide coverage, **γ**, the effective surface energy of the ceria NP, **$\bar{\gamma}$**, the radius of a Ce atom exposed at the (111) surface, **r**, the edge energy, **E_{edge}**, as obtained from the simulations of stepped surfaces (Table **S10**) and the energy contribution of a Ce atom located at a vertex position on the explicit ceria NP, **E_{vert}** (Table **S11**).

Input	Value	Description
**γ**	-0.039 eV/ Å^2	Surface energy of (111)-0.5 ML
**$\bar{\gamma}$**	-0.069 eV/ Å^2	0.5 $\gamma^{(111)\text{-}0.5ML}$ + 0.5 $\gamma^{(100)\text{-}1.0ML}$ as effective surface energy for ceria NP
**R**	2.039 Å	Radius of a Ce atom calculated from its exposed area on (111)
**E_{edge}**	0.303 eV/Å	Edge energy contribution per Å
**E_{vert}**	1.817 eV/Ce	Energy contribution per vertex Ce atom on the CeO_x NP

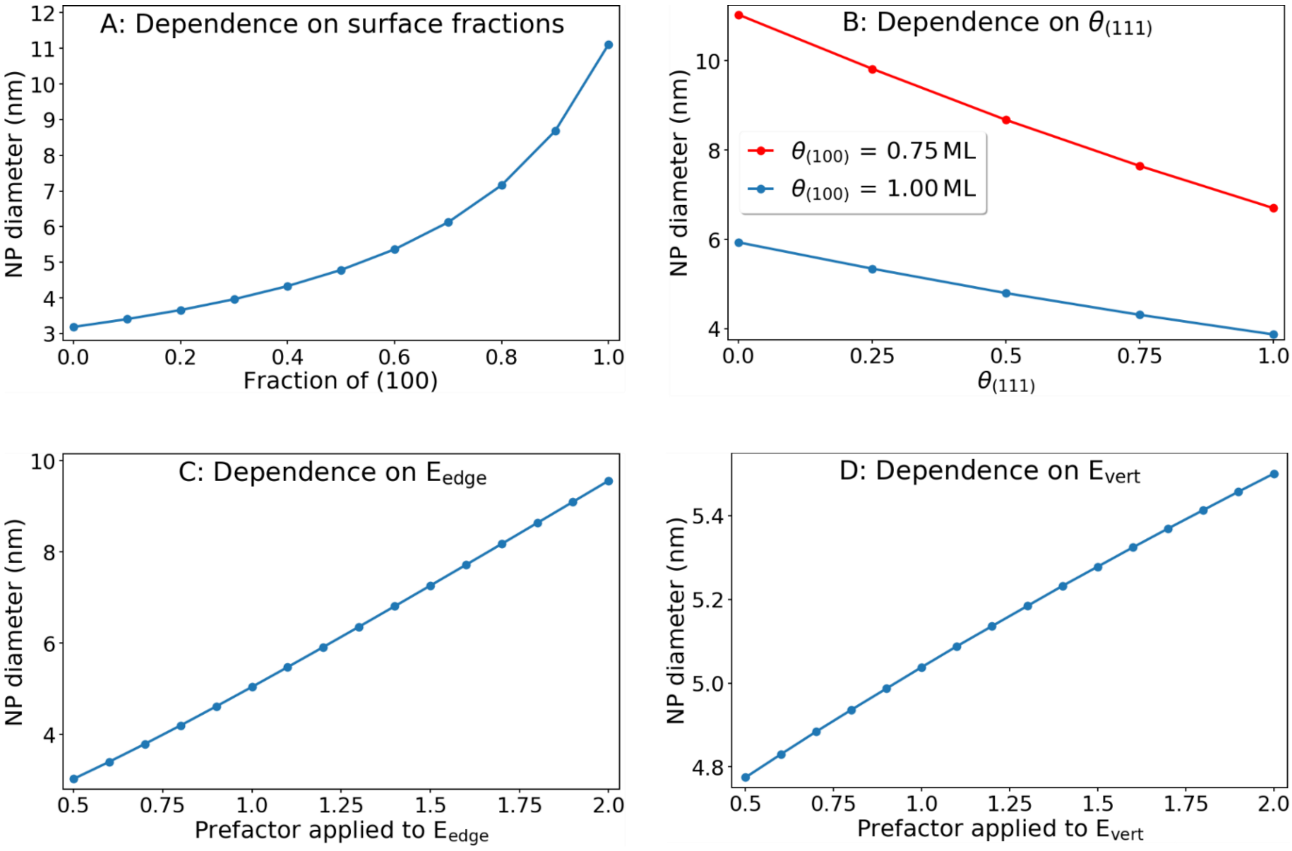

Figure S11. Robustness analysis of the final input parameters in the thermodynamic model.

Table S10. Geometric and calculated properties for the lowest-energy high-index (stepped) surfaces of ceria (the full set is given in Table **S7** and Figure **S9**). The values were obtained for supercells extending one unit cell along the *x* axis and two unit cells along the *y* axis. The tabulated values are the total surface area, $\mathbf{A_{surf}}$, the number of cerium atoms exposed at the surface, $\mathbf{Ce_{surf}}$, the number of surface Ce atoms that are forming the surface step, $\mathbf{Ce_{step}}$, the horizontal length of the step, $\mathbf{L_{step}}$, the surface energy, **γ,** in units of J/m^2 and eV/Å^2, and the energy contribution of the step, $\mathbf{E_{step}}$, in eV. The latter property was obtained as the difference between the total energy of the full calculated surface ($\mathbf{A_{surf}}$ **γ**) and the energetic contributions of the (111)-like surface Ce atoms.

**Surface**	$\mathbf{A_{surf}}$ [Å^2]	$\mathbf{Ce_{surf}}$ ($\mathbf{Ce_{step}}$)	$\mathbf{L_{step}}$ [Å]	**γ** [J/m^2]	**γ** [eV/Å^2]	$\mathbf{E_{step}}$ [eV]
**(112)**	36.931	3 (1)	4.246	0.987	0.062	1.137
**(221)**	45.231	3 (1)	3.285	0.834	0.052	1.217
**(311)**	50.004	4 (2)	4.194	1.133	0.071	1.199

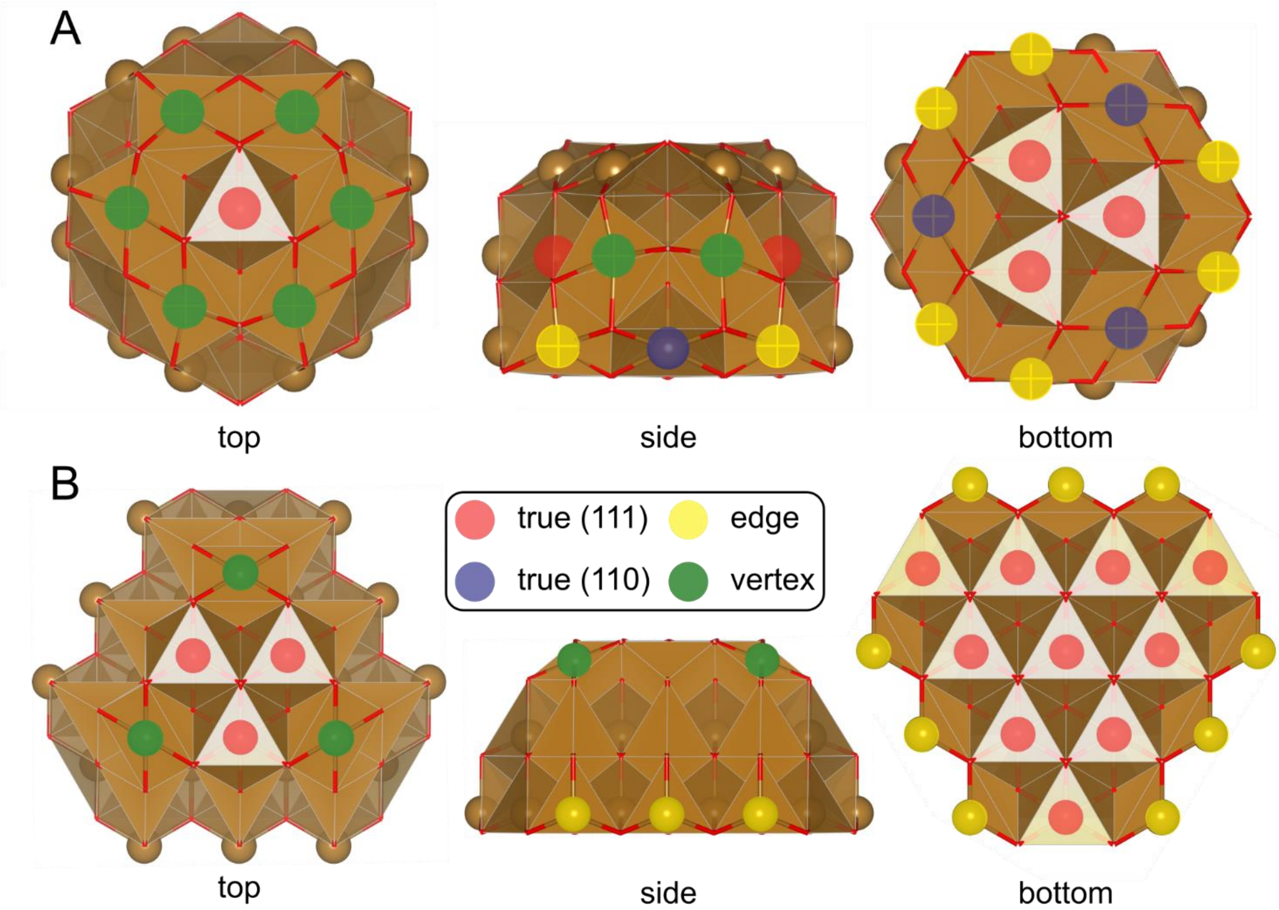

Figure S12. Schematic visualisation of two exemplary, stoichiometric ceria NPs. **A** ($Ce_{31}O_{62}$) is derived from an octahedral structure, exposing exclusively (111) facets (Wulff construction), while **B** ($Ce_{37}O_{74}$) is based on a cubic geometry, exposing (100).

Table S11. Classification of the exposed cerium atoms and calculation of their energy contributions to the energy of the explicitly modelled ceria NP (see Figure **S12-A**). The excess energy of this NP as compared to ceria bulk with the same number of formula units is 37.135 eV. The 28 total exposed surface cerium atoms can be classified as (111)-like, (110)-like, as well as edge and vertex atoms. The surface energies for (111) and (110) were taken from Table **S6**, while the given value of 1.185 eV for edge atoms corresponds to the average of the step energies, $\mathbf{E_{step}}$, that were obtained for the three low-energy high-index surfaces listed in Table **S10**. With these approximations, the remaining total energy contribution of vertex cerium atoms comes out to 21.804 eV, or 1.817 eV per atom.

	**Full CeO_2 NP**	**true (111)**	**true (110)**	**edge**	**vertex**
**Number**	31	7	3	6	12
**E** [eV/Ce]	1.027	0.569	1.413	1.185	1.817
$\mathbf{E_{total}}$ [eV]	37.135	3.982	4.238	7.108	21.804

Note S4. In Figure **S12** and Table **S11** a crude classification of the exposed surface cerium atoms is provided. However, their local geometric environments usually do not strictly conform to a classification derived from the common low-index surfaces. Furthermore, only **A** was employed in the derivation of the thermodynamic model (Note **S2**), as octahedral ceria NPs are more favourable due to the lower surface energy of (111). The inclusion of **B** in Figure **S12**, aims to show that on such nanostructures a high fraction of the exposed surface can be (100)-like and thus provide numerous nanopocket binding sites for Pt: While **A** exposes three such environments (one at each vertex), there are twelve possible positions available on **B**. As both ceria NPs expose (111) at their bottom, they are in full registry with the underlying (111) slab (omitted for clarity). Thus, high-energy oxygen plasma treatment can produce nanostructures on the ceria thin film that are derived from both, octahedral and cubic geometries and provide suitable anchoring points for SA-Pt.

Table S12. Calculated energies (in eV) for Pt-adsorption (referenced against gaseous single-atom Pt) on extended low-index ceria surfaces, CO-adsorption on single-atom Pt and corresponding CO-vibrational frequencies (in cm^{-1}). The calculated vibrational frequencies were scaled using the scaling factor 1.008 based on the experimental (2143 cm^{-1}) and theoretical (2125 cm^{-1}) values obtained for CO in the gas phase.

**Surface**	E_{ads}**(Pt)** [eV]	E_{ads}**(CO)** [eV]	**v(CO)** [cm^{-1}]
**(111)**	-2.798	-2.136	2093
**(111)-0.25** O_2^{2-}	-4.310	-2.327	2081
**(111)-0.50** O_2^{2-} (row)	-3.838	-2.618	2080
**(110)**	-4.991	-0.328	1961
**(100)**	-5.282	-0.712	2065
**(100)-2CO**	-5.282	-2.191	2050, 2121
**(100)-4O**	-6.445	-0.575	2081

Note S5. For the adsorption of Pt on the NP/slab composite system shown in Figure **4** in the main text, most notably coordination at the square-planar nanopockets exposed at the vertices of the ceria NP is about 0.9 eV more favourable than for the same binding site on extended (100). On the other hand, Pt adsorption at the (111)-exposing side facet is by about 0.1 eV more favourable than on extended (111), while adsorption at the (111)-like top facet involves one of the reactive vertex oxygen, thus resulting in increased stabilisation compared to extended (111). Peroxide formation was calculated at the same set of positions on this composite system with the resulting values closely following the results obtained for the extended surfaces, thus not listed here. Lastly, we investigated the effect a peroxide group forming part of the square-planar nanopocket, resulting in a slightly diminished Pt adsorption energy of about -1.55 eV. Similar to the extended slabs (Table **S8**), presence of peroxides does not change this value considerably in the case of the (100) surface (see Note **S3**). Structures with coordinated CO were obtained from the pre-optimized systems containing Pt atom. Removal of one or two ligand oxygen atoms from the initial CO-Pt-4O structure and further optimization yielded the systems “Pt-2O” and “Pt-3O”, as presented in Figure **S13**. Note that optimization of the reference structures only containing Pt leads to a skewed coordination environment in the case of the “3O” system, which might artificially inflate its CO adsorption exothermicity.

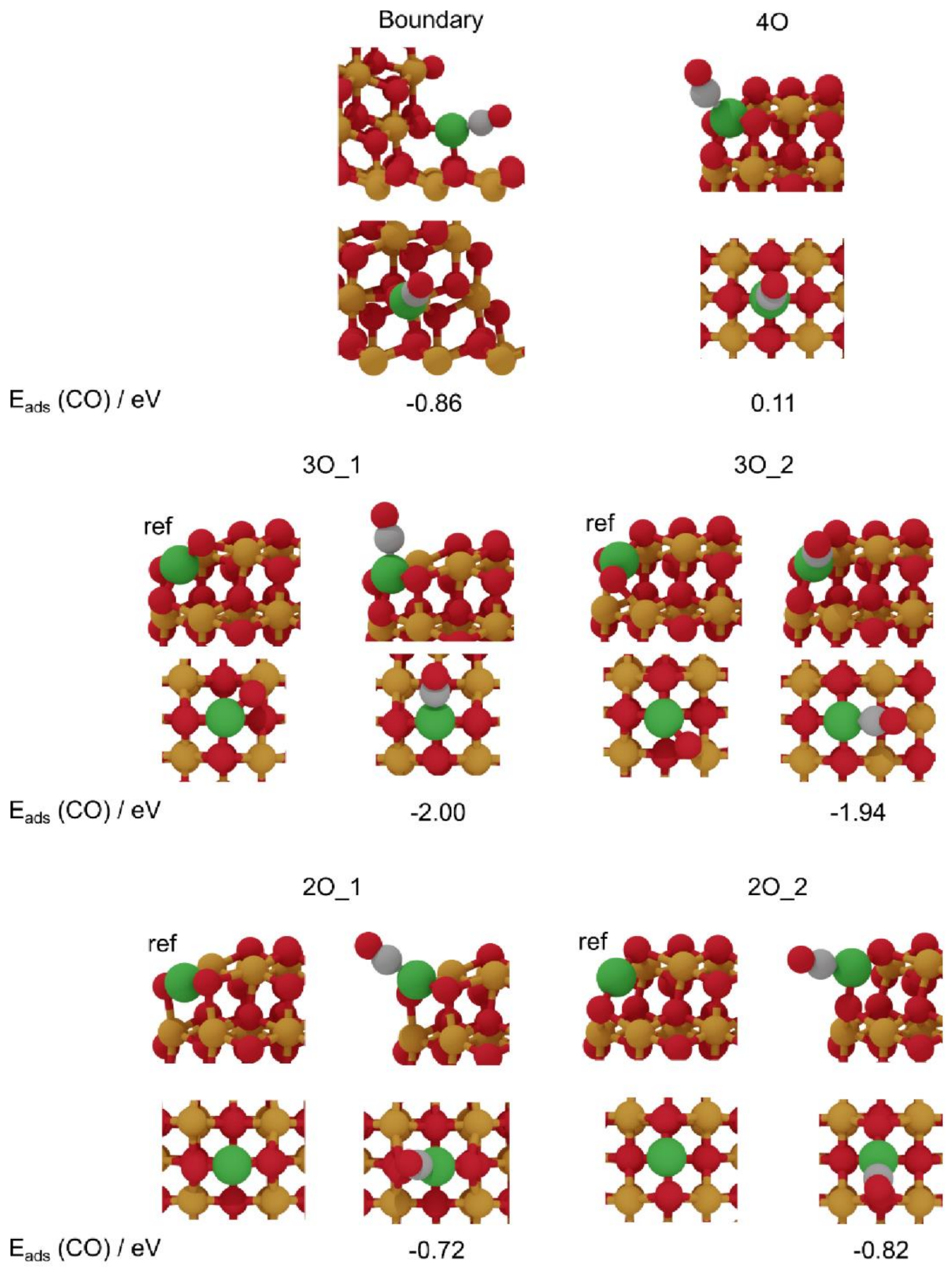

Figure S13. Calculated CO adsorption energies (in eV), for various Pt binding sites on the ceria NP/slab system. Removal of one or two oxygen ligands from the Pt-4O structure leads to several different "2O" and "3O" coordination environments.

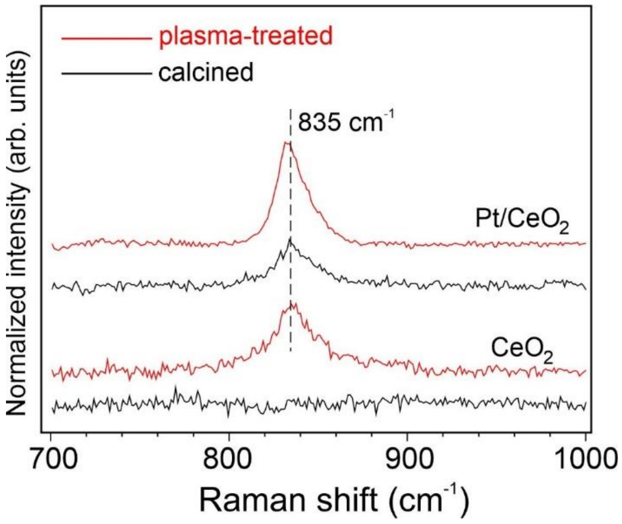

Figure S14. Comparative Raman spectra of CeO_2 and 0.05% Pt/CeO_2 powder catalysts: calcined (in black) and after subsequent treatment in O_2 plasma (in red). The spectra were normalized to the main F_{2g} peak at 465 cm^{-1} (not shown) and are vertically offset for clarity. The peak at around 835 cm^{-1} is characteristic for peroxo species.[9]

References.

[1] aM. Baron, O. Bondarchuk, D. Stacchiola, S. Shaikhutdinov, H. J. Freund, *The Journal of Physical Chemistry C* **2009**, *113*, 6042-6049; bD. R. Mullins, *Surface Science Reports* **2015**, *70*, 42-85.

[2] F. Dvořák, M. Farnesi Camellone, A. Tovt, N.-D. Tran, F. R. Negreiros, M. Vorokhta, T. Skála, I. Matolínová, J. Mysliveček, V. Matolín, S. Fabris, *Nature Communications* **2016**, *7*, 10801.

[3] G. Kresse, J. Furthmüller, *Computational Materials Science* **1996**, *6*, 15-50.

[4] J. P. Perdew, K. Burke, M. Ernzerhof, *Physical Review Letters* **1996**, *77*, 3865-3868.

[5] aP. E. Blöchl, *Physical Review B* **1994**, *50*, 17953-17979; bG. Kresse, D. Joubert, *Physical Review B* **1999**, *59*, 1758-1775.

[6] S. L. Dudarev, G. A. Botton, S. Y. Savrasov, C. J. Humphreys, A. P. Sutton, *Physical Review B* **1998**, *57*, 1505-1509.

[7] N. Pueyo Bellafont, F. Viñes, W. Hieringer, F. Illas, *Journal of Computational Chemistry* **2017**, *38*, 518-522.

[8] Z. Łodziana, N.-Y. Topsøe, J. K. Nørskov, *Nat. Mater.* **2004**, *3*, 289-293.

[9] C. Schilling, A. Hofmann, C. Hess, M. V. Ganduglia-Pirovano, *The Journal of Physical Chemistry C* **2017**, *121*, 20834-20849.

Author Contributions

The manuscript was written through contributions of all authors. S.S. and B.R.C conceived the project and wrote the manuscript; W.W. performed all experiments except STM (carried out by N.B.), STEM (by S.W.C.), and Raman (by M.L.L.). J.G. performed the DFT calculations; J.G., N.L. and N.D. developed the thermodynamic model and wrote the computational part of the manuscript. All authors have given approval to the final version of the manuscript.
